# Hierarchical nano-martensite-engineered a low-cost ultra-strong and ductile titanium alloy

**DOI:** 10.1038/s41467-022-33710-1

**Published:** 2022-10-10

**Authors:** Chongle Zhang, Xiangyun Bao, Mengyuan Hao, Wei Chen, Dongdong Zhang, Dong Wang, Jinyu Zhang, Gang Liu, Jun Sun

**Affiliations:** 1 State Key Laboratory for Mechanical Behavior of Materials, Xi’an Jiaotong University, Xi’an, 710049 People’s Republic of China; 2 Center of Microstructure Science, Frontier Institute of Science and Technology, Xi’an Jiaotong University, Xi’an, 710049 China

**Keywords:** Metals and alloys, Mechanical properties

## Abstract

Due to the low thermal stability of crystallographic boundaries, the grain boundary engineering (GBE) manifests some limits to the fineness and types of microstructures achievable, while unique chemical boundary engineering (CBE) enables us to create a metallic material with an ultrafine hierarchically heterogeneous microstructure for enhancing the mechanical properties of materials. Here, using a low cost metastable Ti-2.8Cr-4.5Zr-5.2Al (wt.%) alloy as a model material, we create a high density of chemical boundaries (CBs) through the significant diffusion mismatch between Cr and Al alloying elements to architecture hierarchical nano-martensites with an average thickness of ~20 nm. For this metastable titanium alloy, the significantly enhanced yield strength originates from dense nano-martensitic interface strengthening, meanwhile the large ductility is attributed to the multi-stage strain hardening of hierarchical 3D α'/β lamellae assisted by equiaxed primary α (α_p_) nodules. The hierarchical nano-martensite engineering strategy confers our alloy a desired combination of strength and ductility, which can potentially be applied to many transformable alloys, and reveal a new target in microstructural design for ultrastrong-yet-ductile structural materials.

## Abstract (web summary)

It is challenging to obtain Ti alloys with ultrafine microstructure owing to the low thermal stability of crystallographic boundaries. Here the authors demonstrate a chemical boundary-based strategy to produce a hierarchical Ti alloy with nano-martensites that has excellent strength and ductility.

## Introduction

Martensitic phase transformation in metastable alloys^1,2^, such as titanium (Ti) alloys^3–6^, steels^7–9^ and multicomponent alloys^10–14^, could achieve one even both of following key benefits: interface hardening due to a dual-phase microstructure (resulting from reduced thermal stability of the high-temperature phase) and transformation-induced hardening (resulting from the reduced mechanical stability of the room-temperature phase)^4,15^. The stress-driven martensitic transformation in alloys, often rendering low yield strength (*σ*_*y*_)^16,17^, enables notable enhancement of ultimate tensile strength (*σ*_*UTS*_), work hardening rate (*θ*) and elongation to failure (*ε*_*f*_), known as the transformation-induced plasticity (TRIP) effect^4,18^. Generally, martensite hardening is captured by the Hall-Petch (like) relationship^19,20^, thus it is anticipated to engineer nano-martensites in microstructures so as to strengthen and ductilize alloys for excellent mechanical properties.

High specific strength Ti alloys that can be engineered to high *σ*_*y*_ with large *ε*_*f*_ are important structural materials for light-weighting^3,4,15^. In principle, the extraordinary mechanical properties of duplex Ti alloys are tuned by modulating the quantity or arrangement of grain boundaries (GBs) and hetero-phase boundaries (PBs), e.g., α/β interfaces, which are planar discontinuities in alloys^21–23^. The duplex microstructure composed of equiaxed primary α (α_p_) nodules and colonies of secondary α (α_s_) lamellae embedded in the β matrix endows Ti alloys with a well-balanced property profile^5,23^. In addition to the diffusion (β-to-α) transformation, PBs can be introduced via a diffusionless displacive (β-to-α') transformation depending on the cooling rate and chemical composition^5,15,24,25^. The density of PBs or the size of martensites α' can be tuned by the density of chemical boundaries (CBs) defined by a sharp discontinuity of at least one elemental concentration inside a lattice-continuous region^26^, because CBs at high temperature can restrict the growth of martensites for microstructural refinement. Recent findings in Ti-4Mo and Ti-6Al-4V alloys have demonstrated that when the microstructure consists of micron- and submicron-scaled α' and primary α phases, they exhibit notably enhanced tensile properties^5,27^. However, metastable duplex Ti alloys (containing micro-martensites), e.g., Ti-6Al-4V alloys often suffer from relatively low *σ*_*y*_ on the order of 1100 MPa or even less^19,28^. It is desirable, therefore, to devise a nano-martensite-strengthened Ti alloy with high yield strength *σ*_*y*_ and ductility via the CB engineering (CBE) strategy.

In this work, we tailor the density of CBs at high temperature by tuning the β-stabilizer Cr concentrations, thereby regulating the subsequent phase transformation behavior of low-cost Ti-*x*Cr-4.5Zr-5.2Al (*x* = 1.8, 2.3 and 2.8 wt.%) alloys (Refer to Supplementary Figs. 1, 2 for the detailed composition design of our nano-martensitic Ti alloys). Utilizing the CBE strategy, we create the finest size of nano-martensites reported by far to realize the highest yield strength of martensitic Ti alloys with great ductility. Compared with the forged alloys undergoing air cooling (AC, with *σ*_*y*_ ~981 MPa and *ε*_*f*_ ~22.8%), this hierarchically structured WQ Ti-2.8Cr-4.5Zr-5.2Al alloy with ~20 nm-thick nano-martensites exhibits ultra-high strength *σ*_*y*_ ~1266 MPa with great ductility *ε*_*f*_ ~12.6%.

## Results

### Hierarchical microstructures of Ti-Cr-Zr-Al alloys

**Fig. 1:**
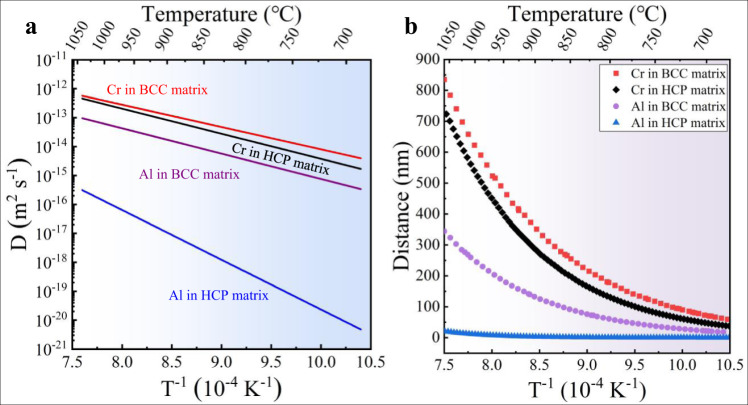
Diffusion coefficient and distance. **a** Temperature dependent diffusivity *D* of Cr and Al elements in BCC-Ti and HCP-Ti matrix, respectively. **b** Temperature dependent diffusion distance *L* per second of Cr and Al elements in BCC-Ti and HCP-Ti matrixes, respectively.

The formation of CBs at high temperature (e.g., above the phase transformation temperature) during either the AC or the WQ process is determined by the difference in the diffusion distance *L* of alloying elements at a time period *t* that can be simply calculated via $L=\sqrt{{Dt}}$, where the diffusion coefficient *D* at a specific temperature *T* (in Kelvin) can be measured in the Arrhenius diagram of element diffusion in the BCC or HCP-Ti matrix^29–31^, see Fig. 1a. The Cr element has a much larger *L* or *D* (~1.56 × 10^−13^ m^2^ s^−1^ in β-Ti) than that of Al (with *D* ~ 2.01 × 10^−14^ m^2^ s^−1^ in β-Ti) at 1193 K (920 °C) (Fig. 1a, b). Therefore, the obvious difference in *L* between Cr and Al inevitably leads to the formation of a high density of nonequilibrium CBs before phase transformation in the high-temperature β phase. It should be pointed out that although the diffusion distance of the Cr element decreases during quenching, it is still much higher than that of the Al element, see Fig. 1b. These CBs divide the coarse β-grains into a large number of ultrafine even nanoscale domains alternately enriched or depleted in Al (and simultaneously depleted or enriched Cr). Once formed, CBs act as barriers restricting subsequent phase transformation within nanoscaled domains enriched in Cr or depleted in Al, while the nanoscaled Al-enriched or Cr-depleted domains serve as the fertile for the formation of dense nano-precipitates. Thus, in addition to GBs, the high density of CBs existing inside the β-grains can act as a new feature to realize microstructural architecture.

**Fig. 2:**
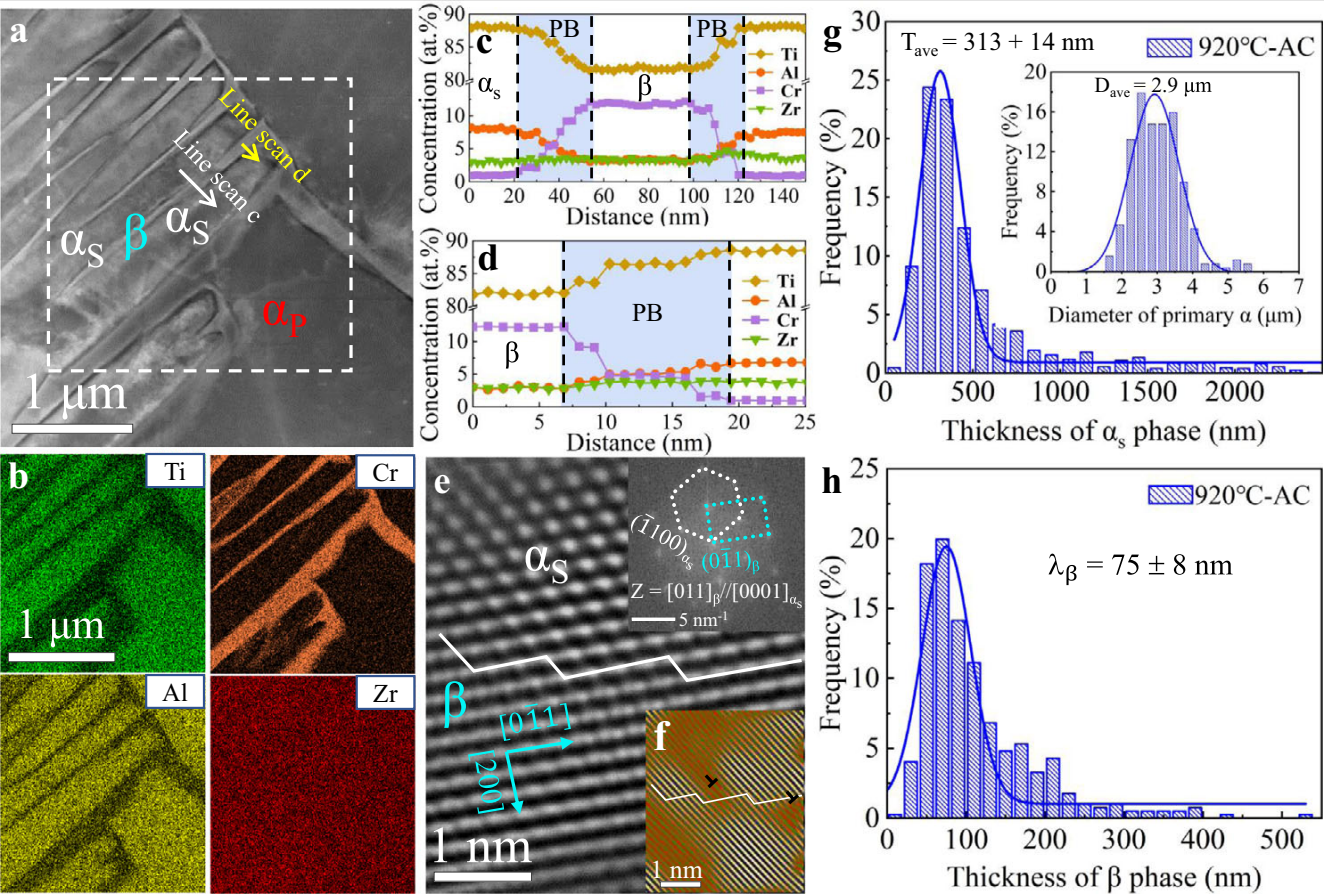
Microstructural characterization of the Ti-2.8Cr-4.5Zr-5.2Al alloys after heat treatment: AC. **a** An scanning TEM (STEM) image showing the β_trans_ (α_s_ + β) + α_p_ structure. **b** The energy dispersive spectroscopy (EDS) maps of the identical region marked in **a** showing the distribution of Ti, Cr, Al, and Zr. **c**, **d** The EDS composition profiles showing the α_s_/β microstructure. **e** High-resolution (HR) TEM and corresponding fast Fourier-transform (FFT) images showing the semi-coherent β/α_s_ interface, associated with misfit dislocations. **f** [2$\bar{1}$1]_β_ lattice fringes obtained by inverse FFT filtering showing misfit dislocations at α_s_/β interface. **g** The distribution of α_s_ precipitates as well as α_p_ particles (inset) in the AC sample. **h** The distribution of β lamellae for the present AC alloys.

**Fig. 3:**
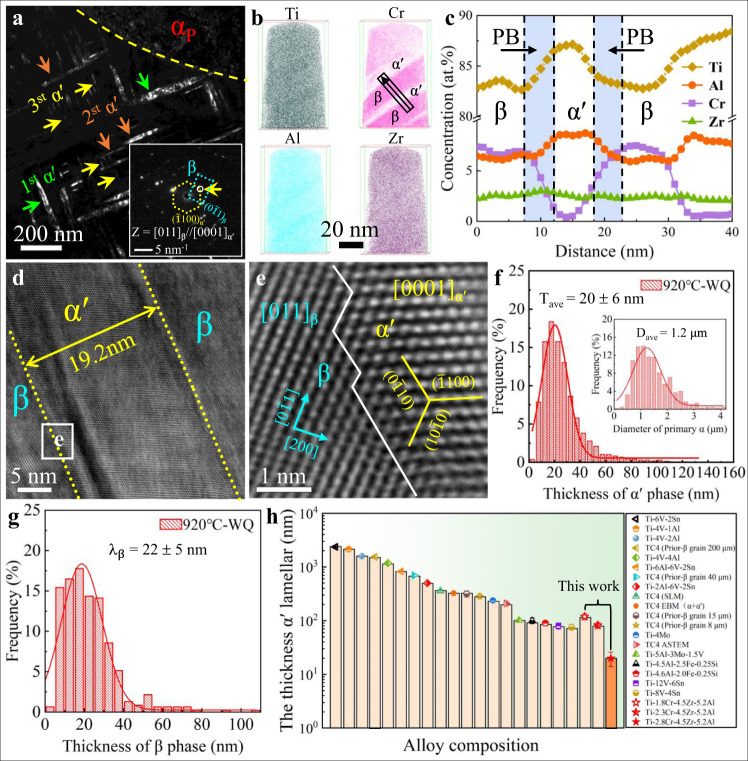
Microstructural characterization of the Ti-2.8Cr-4.5Zr-5.2Al alloys after heat treatment: WQ. **a** The dark field TEM image showing the microstructure consisting of β_trans_ (α' + β) + α_p_. **b**, **c** APT characterization showing element distributions in Ti-Cr-Zr-Al alloys. **d** An HR-TEM image showing the nanometer size α', ~19.2 nm. **e** An HR-TEM image of the corresponding region of **d** showing the β/α' phase boundary. **f**, **g** Distribution of the α' and β phases in the WQ sample. The inset in **f** is the distribution of α_p_ particles in the WQ sample. **h** A comparison of the α' thickness for the present Ti-*x*Cr-4.5Zr-5.2Al (*x* = 1.8, 2.3, and 2.8) alloys and other reported martensite Ti alloys, including Ti-4Mo^5^, Ti-5Al-3Mo-1.5V^37^, SLM-TC4^25, 38^, As-HIP′ed TC4^6^, TC4 (prior-β grain)^19^, Ti-V-(Al, Sn) series^39^, and Ti-V-Sn series^40^. Error bars represent standard deviation.

To utilize the CBs for creating a refined microstructure, a solution temperature of 920 °C lower than the β-transus temperature (T_β_) ~ 955 °C was selected for 1 h to homogenize the chemical composition of both the primary α (α_p_) and β-parent phases in our duplex Ti-2.8Cr-4.5Zr-5.2Al alloys, and the thermomechanical processes see Supplementary Fig. 3. When subsequent cooling, i.e., AC and WQ, were respectively performed, and the α_p_ phase was retained while the β phase suffered from different phase transformations associated with different morphologies (see Fig. 2 and 3 for AC and WQ samples, respectively), depending on the phase stability of the local regions, which was influenced by the cooling rate-related CBs^15,26^.

Under the AC condition, the chemical gradients of CBs decrease with decreasing temperature before phase transformation, and the concentrations of alloying elements in the matrix have sufficient time to homogenize. Based on the CBE viewpoint^26^, sufficient time for atomic long-range diffusion in β-grains allows for a wide chemical discontinuity in the spatial distribution of alloying elements of Cr and Al, resulting in the formation of nonequilibrium wide CBs with a low density. Once the structural transformation occurs, these wide CBs would coexist with the crystallographic lattice discontinuity, rendering the formation α_s_/β PBs at low temperature. Figure 2a, b shows the hierarchical microstructure of AC Ti-Cr-Zr-Al alloys consisting of microscaled equiaxed α_p_-grains and submicron-scaled α_s_ and nanoscaled β lamellae and the distribution of alloying elements. Indeed, the displacive β-to-α transformation takes place mainly in Al-enriched (and simultaneously Cr-depleted) domains inside each β-grain and thus the secondary α-phase (α_s_) precipitates in AC Ti-Cr-Zr-Al alloys, see Fig. 2c. Subsequently, these α_s_ precipitates grow up with decreasing temperature until the chemical gradient can stop their propagation and thickening by a local reduction in driving force^26^, rendering the formation of submicron-scaled α_s_ laths and wide α_s_/β PBs in the range of ~25 to 35 nm, see Fig. 2c, d. An High-resolution transmission electron microscope (HR-TEM) image shows the semi-coherent α_s_/β phase interface^32,33^ decorated with misfit dislocations (Fig. 2e, f), and the α phase maintain the typical Burgers orientation relationship (BOR) with the β phase, i.e., (0001)_α_//(011)_β_ and [$\bar{1}$100]_α_//[$\bar{1}$1$\bar{2}$]_β_. Given the experimental scatter in energy dispersive spectroscopy (EDS) data, the element distribution between the as-forged and AC samples is nearly identical, see Supplementary Fig. 6 and Supplementary Table 1. These results imply that the continuous transformation of α_s_, accompanied with the growth of α_s_ (and migration of PBs), leads to each phase of AC samples is in equilibrium at ambient temperature after sufficient diffusion. Furthermore, this bimodal α-structure was quantified by measuring their dimensions and fractions of both α_p_ and α_s_ precipitates, see Fig. 2g. It appears that the average diameter D_ave_ and the volume fraction F_v_ of the α_p_-phase are ~2.9 μm and ~30%, respectively, while these values of the α_s_-phase are T_ave_ ~313 ± 14 nm and F_v_ ~52.4%, respectively. The average thickness of β-lamellae is *λ*_β_ ~75 ± 8 nm, see Fig. 2h.

By contrast, the WQ process triggers intense concentration fluctuations to form dense narrow CBs in the β-parent (above the phase transformation temperature), which will be frozen at relatively low temperature due to the insufficient diffusion time and the fast cooling rate^5,15^. These CBs can divide each β-grain into a large number of nanodomains for martensitic transformation and strongly restrict the growth of nano-martensites in our WQ Ti alloy. However, these CBs are not preserved at room temperature as well, because diffusionless martensitic transformation with fast speed can complete instantaneously, rendering these CBs with notable concentration gradients transform into the α'/β PBs with crystallographic lattice discontinuity. In this scenario, these α'/β PBs in WQ Ti alloys mainly stem from the prior CBs existed above the phase transformation temperature, similar to the case of a low carbon steel^26^. Thus, for the sake of simplicity, the density of CBs is considered to be almost equal to that of PBs. Figure 3a shows the hierarchical microstructure of WQ Ti-Cr-Zr-Al alloys consisting of microscaled equiaxed α_p_-grains and nano-scaled α' and β nanolamellae. According to their sizes and orientations, these α' martensites (with the lattice parameters of *a* = 0.293 nm, and *c* = 0.466 nm, Supplementary Figs. 4, 5) can be divided into primary, secondary and even tertiary nanomartensites (Fig. 3a). As a result, these 3D α'-nanolamellae intersect with each other and effectively divide the β-parent into a large number of β nano-domains. These nanomartensites maintain a Burgers orientation relationship^5^ with the β-parent as (0001)_α'_//(011)_β_ and [$\bar{1}$100]_α'_//[$\bar{1}$1$\bar{2}$]_β_, as verified by the selected area electron diffraction (SAED) in Fig. 3a. These fine α'-plates result in the broadening of their diffraction peaks in the XRD pattern in Supplementary Fig. 5.

The atom probe tomography (APT) analysis for the WQ Ti-Cr-Zr-Al sample shows that the neutral Zr element is homogeneously distributed throughout the microstructure, while notable compositional partitioning of Cr, Ti and Al takes place between α' and β phases, where the former is enriched by the α-stabilizer (Al), while the latter is enriched by the β-stabilizer (Cr), see Fig. 3b, c. The corresponding APT maps and 1D concentration lines of Cr and Al in the WQ samples confirm a narrow chemical transition region with a width of ~4 nm for the α'/β PBs (Fig. 3c), much narrower than that of in their AC siblings. This is because the rapid cooling strongly limits the diffusion length of alloying elements, resulting in a high density of sharp prior CBs in these metastable β-grains that can significantly refine the size of nano-martensites. Figure 3a shows that the martensitic lamella interiors are decorated by dislocations rather than twins, implying that the produced nanomartensites actually belong to dislocation-structured martensites. An HR-TEM image shows the nanoscaled α' lamellae (~19.2 nm), see Fig. 3d. In particular, the interfacial structure between α' and β is fully coherent (Fig. 3e), which is different from the conventional steel associated with semi-incoherent or incoherent interfaces between the matrix and martensites decorated with misfit dislocations^34–36^. Moreover, in WQ Ti-Cr-Zr-Al alloys, the average thickness T_ave_ and volume fraction F_v_ of α'-lamellae are ~20 ± 6 nm and ~58.7%, respectively, see Fig. 3f. The average thickness of β-lamellae is only *λ*_β_ ~ 22 ± 5 nm, see Fig. 3g. Obviously, this is a record fine α'-lamellar thickness among all martensitic Ti alloys reported to date, see Fig. 3h.

### Extraordinary mechanical properties

**Fig. 4:**
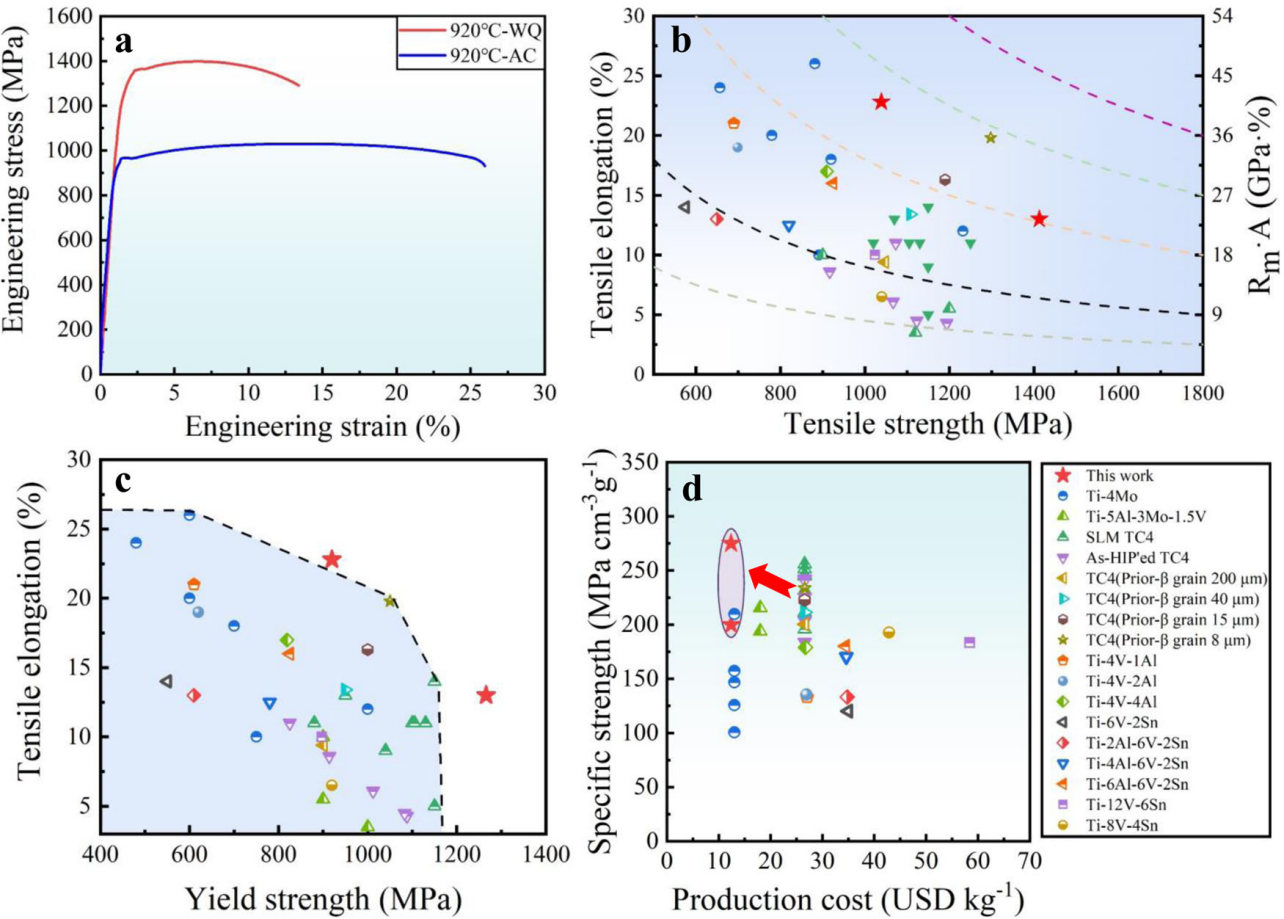
Room-temperature tensile properties of the present Ti-2.8Cr-4.5Zr-5.2Al alloys compared with other high strength α'/β-Ti alloys in the literature. **a** The engineering stress-strain curves. Comparison of **b** the tensile strength and total elongation, and **c** the yield strength and total elongation of the present Ti alloy to other reported high strength α'/β-Ti alloys to date. **d** Comparison of the specific yield strength and production cost of the present Ti alloy to other reported high strength α'/β-Ti alloys.

Figure 4a shows representative tensile engineering stress-strain curves of both AC and WQ Ti-Cr-Zr-Al alloys. It appears that the AC samples initiate to yield at a stress of ~920 MPa (*σ*_*y*_) and have an ultimate tensile strength (*σ*_*UTS*_) of ~1039 MPa along with a large ductility (*ε*_*f*_) of ~22.8%. In contrast, the WQ samples manifest a dramatically enhanced strength of *σ*_*y*_ ~1266 MPa and *σ*_*UTS*_ ~ 1413 MPa associated with good ductility *ε*_*f*_ ~12.6%. Figure 4b, c respectively plots the elongation to fracture *ε*_*f*_ that characterizes the resistance to damage against the strength *σ*_*UTS*_ as well as *σ*_*y*_ of our Ti-Cr-Zr-Al alloy, together with those reported high-performance α'/β-Ti alloys, including Ti-4Mo^5^, Ti-5Al-3Mo-1.5V^37^, SLM-TC4^25,38^, As-HIP′ed TC4^6^, TC4 (prior-β grain)^19^, Ti-V-(Al, Sn) series^39^, and Ti-V-Sn series^40^. Obviously, our Ti-Cr-Zr-Al alloy exhibits a better combination of strength and ductility, in comparison to other duplex α'/β-Ti alloys reported to date. In particular, the unprecedented high strength (*σ*_*y*_ ~1266 MPa and *σ*_*UTS*_ ~ 1413 MPa) sets the present Ti-Cr-Zr-Al WQ alloy apart from all the reported α'/β-Ti alloys, which in general have either low strength or low ductility (or both low). In other words, the WQ alloy displays the highest strength and tensile ductility, i.e., an excellent combination of strength and ductility. Indeed, the product of *σ*_*UTS*_ and *ε*_*f*_, often as a good indicator of fracture toughness, in the range of 18 GPa% to 27 GPa% or the present Ti-Cr-Zr-Al alloys is much higher than most of the reported Ti alloys mentioned above. Moreover, the specific yield strength (SYS) *vs*. the production cost (the average metal cost of alloying elements, see Supplementary Table 2) of our Ti-Cr-Zr-Al alloy as well as other reported duplex α'/β-Ti alloys is displayed in Fig. 4d. It seems that our alloy highlights the highest SYS and the lowest production cost simultaneously in comparison to reported ones. Given the facile thermomechanical processing and simple heat treatment, it is expected that this low cost, ultra-strong and ductile Ti-Cr-Zr-Al alloy could broaden its applications in more industries.

## Discussion

### Formation of hierarchical nano-martensite microstructure

Often, a high cooling rate can strongly restrict the diffusion of atoms, resulting in the formation of micron- and submicron-martensites in metastable Ti alloys^5^. For the present Ti-Cr-Zr-Al alloy, the formation of dense nanomartensites originates from the CBs that represent a notable chemical discontinuity inside a continuous lattice region during WQ. In other words, the coupling effect of Cr and Al diffusion leads to a novel hierarchically heterogeneous microstructure consisting of nanomartensites and residual β nanolamellae in each original β-grain. To fully understand the formation of hierarchical nano-martensites in this work, phase field modeling (Supplementary Note 5) was employed to elucidate the underlying mechanism(s). It should be noted that our simulations only focused on the intragranular microstructure and no consideration of GBs or other defects.

**Fig. 5:**
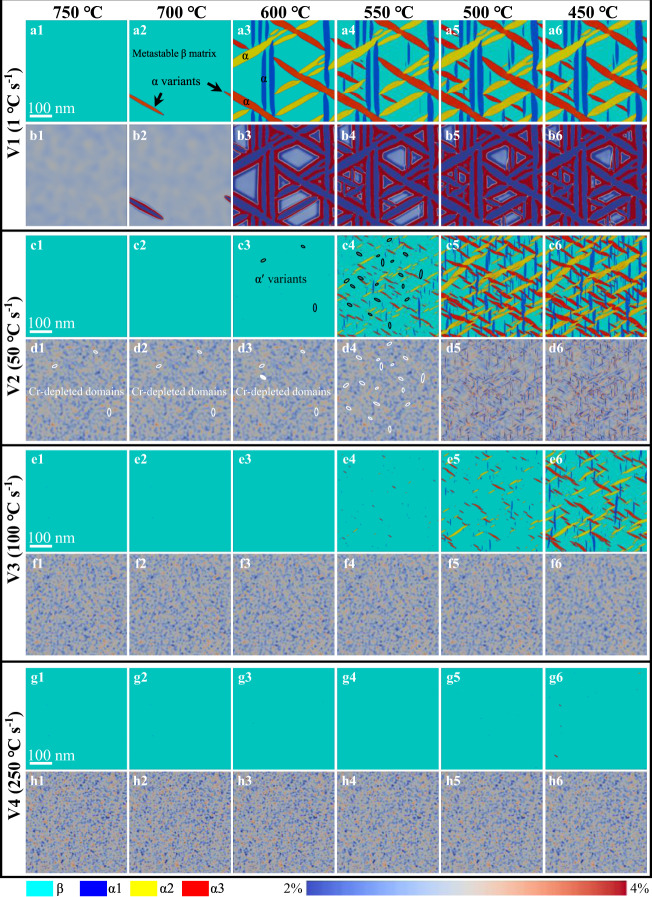
The evolution results of the concentration field and composition field are output every 50 °C from 750 °C to 450 °C under different cooling rates. **a1**–**a6**, **c1**–**c6**, **e1–e6**, **g1**–**g6** The corresponding structure fields after different cooling rates. Light blue color represents the β phase, and dark blue, yellow, and red colors describe three variants of α precipitates. **b1**–**b6**, **d1**–**d6**, **f1**–**f6**, **h1**–**h6** The composition fields after different cooling rates, light blue and near red stand for the Cr-depleted and Cr-enriched domains, respectively. The color bar refers to the Cr concentration (wt.%) and different colors distinguish the Cr-depleted domains (light blue) and Cr-enriched domains (near red).

For a more precise observation of the microstructure evolution during the cooling process, we displayed in Fig. 5 the structure field and concentration field every 50 °C from 750 °C to 450 °C (temperature below which the results can be found in Supplementary Fig. 8). Indeed, if the cooling rate is slow (V1 for AC), there is sufficient time to homogenize the distribution of solutes associated with the reduction in chemical fluctuations with decreasing temperature, see Fig. 5b1, b2. At this cooling rate, the precipitation of α phase is dominated by the classical nucleation and growth mechanism, see black arrows in Fig. 5a2. If the cooling rate is faster (take V2 as an example for WQ), intense concentration fluctuations (Cr-depleted domains were marked via white circles) existed at 920 °C are still maintained at 700 °C, Fig. 5d1, d2. When the temperature decreases to 600 °C, the α'-phase nucleus (structural transformation) are more easily generated at these Cr-depleted domains, see black circles in Fig. 5c3, c4. These Al-enriched or Cr-depleted nanodomains serve as the fertile for the formation of nano-martensites, while the CBs act as barriers restricting subsequent phase transformation within nanodomains enriched in Cr or depleted in Al, see Fig. 5c4, d4. Similar phenomenon is observed at higher cooling rates, Fig. 5e–h. The calculated microstructure with increasing the cooling rate from 1 °C s^−1^ to 250 °C s^−1^ are displayed in Supplementary Fig. 8, which proves the sizes and types of α and β are closely related to the cooling rate. Specifically, the average size of nano-martensites from the simulation at fast cooling rate (V2: 50 °C s^−1^) corresponds well to that of the WQ Ti alloys.

**Fig. 6:**
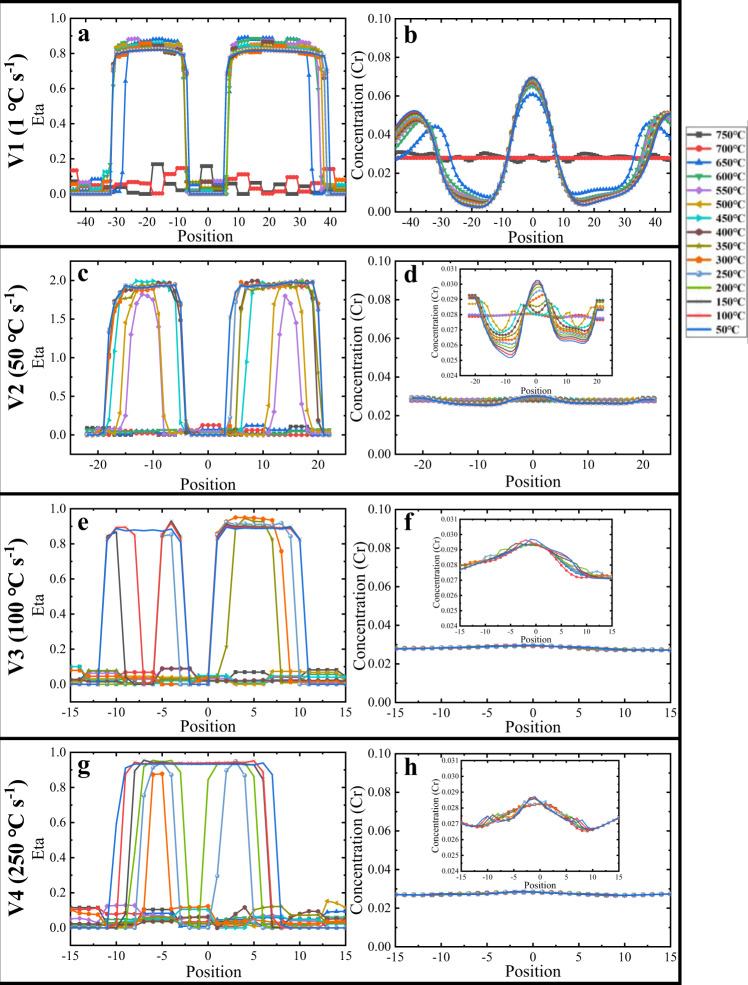
Evolution of structural order parameters and concentration distribution (Cr) with temperature along the center of supercritical nuclei produced by different nucleation mechanisms at different cooling rates. Evolution of the structural order parameter (**a**) and concentration (**b**) of the α nucleus formed by the conventional nucleation and growth mechanism. Evolutions of the structural order parameter (**c**) and concentration (**d**) of the α' nucleus formed by the martensite mechanism. Evolution of structural order parameters (**e** and **g**), and concentrations (**f** and **h**) of α' nucleus formed by the martensitic transformation mechanism.

Further, more obvious evidences indicate that different cooling rates correspond to different nucleation mechanisms according to the evolution of structural field and concentration field (the distribution of Cr element) along supercritical nucleus, as shown in Fig. 6. Figure 6a, b respectively shows the evolution of the structural field and concentration field along a core of α precipitates formed by the classical nucleation mechanism, in which transformation of the structure field is complete (order parameter: Eta > 0.8) and the concentration field reaches the equilibrium states simultaneously. In contrast, the structure field and concentration evolution of the α'-phase nucleus generated by V2 shown in Fig. 6c, d, respectively, prove the existence of the martensite mechanism, where concentration diffusion and structural transformation are not synchronized. This is similar to the main characteristic of the pseudospinodal mechanism, that is, the structure transformation is complete and rapidly while the concentration keeps evolving towards equilibrium^41,42^. However, due to the continuous increase in cooling rates, the concentration field will maintain the initial distribution. Therefore, the microstructure formed by rapid cooling, most of its composition change originates from the CBs with notable concentration gradients at high temperature, can be called the martensitic phase.

### Mechanisms for ultra-high strength

**Fig. 7:**
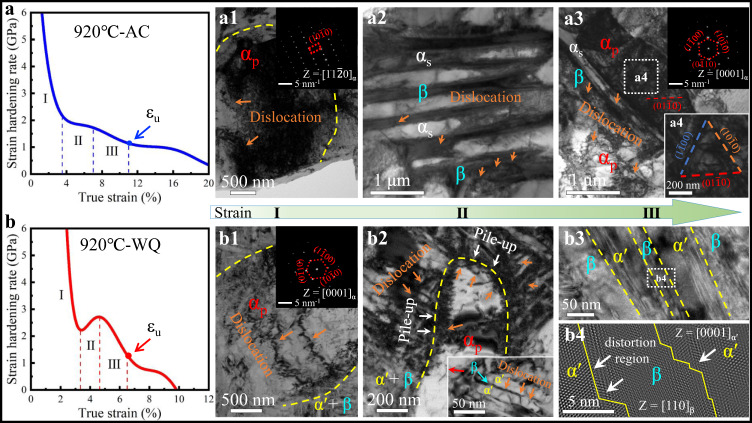
Strain hardening rate curves and the underlying mechanisms for the present AC and WQ Ti alloys. **a**, **b** Strain hardening rate curves for AC and WQ samples, respectively. **a1**–**a4** Tensile tests were interrupted at three critical stages for the AC Ti alloy. **a1** A TEM image showing dislocations in the α_p_ phase, stage I. **a2** A TEM image shows confined dislocations in β layers, stage II. **a3**, **a4** A TEM image shows co-deformation between β, α_s_ and α_p_, stage-III. **b1**–**b4** Tensile tests were interrupted at three critical stages for the WQ Ti alloy. **b1** A TEM image shows dislocations in the α_p_ phase, stage I. **b2** A TEM image shows that hetero-deformation promotes dislocation-interface interactions and dislocation nucleation within the α_p_ phase, stage-II. The inset shows dislocation-interface interactions at α'/β interfaces. **b3**, **b4** The bright field and HR-TEM images showing dislocations can transmit across the α'/β interface, leading to localized shearing, stage-III. The uniform elongation (*ε*_*U*_) is determined based on Considère’s criterion.

A combination of high strength and good ductility has been demonstrated in our designed Ti-2.8Cr-4.5Zr-5.2Al alloy. In particularly, the ultra-high strength (*σ*_*y*_ ~ 1266 MPa and *σ*_*UTS*_ ~ 1413 MPa) sets the present WQ nano-martensitic Ti alloy apart from all the previously reported α'/β-Ti alloys. The deformation mechanism and microstructural morphologies are two key factors in determining alloy deformability^5,43,44^. In the deformed microstructure, there is only a large number of dislocations and dislocation entanglements, and no deformation twins are observed. It has been verified that prismatic slip is preferentially activated, especially for slip systems with high Schmid factors^45^. With respect to the microstructure morphology, these α_p_ grains with larger sizes have relatively longer effective slip lengths, thereby a lower deformation resistance for α_p_ with respect to α_s_ or α' lamellae^6,23^. Upon loading, the soft α_p_ precipitates plastically deform first (Supplementary Fig. 9), and they cannot deform freely and are constrained by the surrounding hard α'/β nanolamellae structure in our WQ samples, see Fig. 7b1. While for the β-transus microstructure, the ultrafine 3D martensitic network isolates each β-grains in submicron domains, which strongly restricts dislocation pile-up at α'/β PBs. This hinders slip propagation from β to α' lamellae, thus a higher stress is required to initiate macroscopic yielding of the β-transus microstructure. Therefore, the yielding of CBE-processed martensitic Ti alloys is governed more by the hard nanomartensite frame rather than by the soft α_p_ phase. As a result, the strength discrepancy between α_p_ and the β-transus microstructure would cause severe strain incompatibility and thus strain gradients, which is accommodated by geometrically necessary dislocations (GNDs) piled up against the PBs^46,47^, see white arrows in Fig. 7b2. Similarly, there is a notable strain gradient between soft residual β and hard α' nano-lamellae in the WQ sample. It is the root of the large heterogeneous deformation-induced stress that contributes to the measured ultra-high strength, even the good strain hardening capability and thus the uniform elongation of our nanomartensitic Ti alloy^46–48^.

In general, the strengthening mechanisms of nanomartensitic Ti alloys usually include solid solution strengthening, dislocation strengthening (Supplementary Figs. 10, 11), precipitation strengthening and boundary/interface strengthening. In the present α'-martensitic Ti alloys, the characteristics of laths, blocks and packets are obvious, and the packet size or block size is often regarded as the effective grain size for GB strengthening^5^. The high strength is predominantly produced by the high-density fine-dispersed α'-martensite platelets. A simple strengthening model is employed to estimate the various contributions to the yield strength *σ*_*y*_ of AC and WQ Ti alloys, which is in good agreement with the experimental results of AC and WQ samples, as marked in Supplementary Fig. 12. The contribution of α'/β interface strengthening is ~421 MPa, verifying that the high yield strength of WQ alloys indeed comes from hierarchical nano-martensite interfaces. For the detailed calculation, please refer to the theoretical calculation of strength in the Supplementary Note 7.

### Origin of high ductility

High strain hardening capability is the premise of large uniform elongation for ductile alloys. Figure 7a, b show the strain hardening rate (*θ*) as a function of the true strain for AC and WQ Ti-Cr-Zr-Al alloys, respectively, both of which have three distinguishable stages. The AC sample shows *θ* first drops quickly in stage I, and then almost maintains a *θ-*plateau in stage II. With increasing strain, *θ* slowly decreases in stage III. By contrast, *θ* initially descends to 2.2 GPa in the WQ sample, followed by a slight increase to 2.75 GPa at a strain of ~3.2%, whereas *θ* again decreases after a strain of ~4.6%. Moreover, the WQ Ti alloy always exhibits a higher *θ* than that of the AC sample before necking. Next, the dynamic evolution of the deformation substructure was uncovered to elucidate the different mechanisms for the work hardening capability of the WQ and AC samples.

In stage-I, the sharply reduced *θ* is caused by dislocation slip in the soft α_p_ phase for both stretched AC and WQ Ti-Cr-Zr-Al alloys^23,49^, see Fig. 7a1, b1. In stage-II, the nearly *θ-*plateau mainly originates from the activation of confined dislocation slip in the β-transus microstructure with lamellar structure in AC samples (Fig. 7a2), similar to that of multilayered materials^50^. For the WQ sample, the hetero-deformation triggers abundant GNDs pile-up against PBs with high hetero-deformation induced stresses^46^, leading to increased strain hardening rate (see white arrows in Fig. 7b2). At the same time, the increased stress not only promotes dislocation-interface interactions such as dislocation transmission across the interface (as marked by orange arrows in the inset of Fig. 7b2), but also facilitates dislocation nucleation from sources such as boundaries/interfaces, as indicated by the orange arrows inside the primary α_p_ phase in Fig. 7b2. These processes are beneficial for dislocation accumulation, thereby enhancing *θ* of WQ alloys^47^. In stage-III, although co-deformation between the two phases occurs accompanied by dislocation accumulation near the interface, dislocation slip is mainly localized on different slip systems inside α_p_, resulting in a slightly decreased *θ* in the AC samples, see Fig. 7a3, a4. In contrast, under such high applied stresses, dislocations can transmit across coherent α'/β interfaces, as verified by the severely distorted and curved α'/β interfaces in Fig. 7b3, b4. This leads to localized shearing of the β-transus microstructure with limited dislocation storage^48^, thus sharply reducing *θ* in the WQ sample. It should be pointed out that although the *θ* of the WQ sample decreases in stage III, it is still higher than that of the AC sample at this stage.

**Fig. 8:**
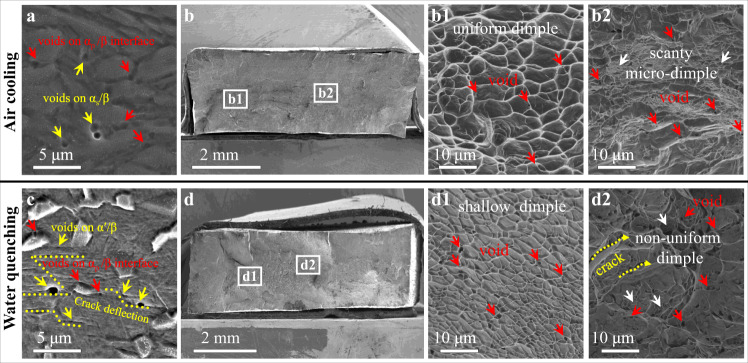
Typical SEM images of damage-evolution mechanisms for the present AC and WQ samples. **a**, **b** The WQ sample. **a** An SEM image of the fractured surface shows voids on the α_p_/β and α_s_/β interfaces. **b** Projection of the entire fracture surface of the AC sample. **b1** A magnified image of the fracture surface showing uniform dimples and voids. **b2** An SEM image shows some voids and small dimples in the center of the fracture surface, but no long cracks. **c**, **d** The AC sample. **c** An SEM image of the fractured surface shows crack propagation and deflection at the α'/β interfaces. **d** Projection of the entire fracture surface of AC sample. **d1** A magnified image of the fracture surface showing shallow dimples and voids. **d2** An SEM image shows that micro-voids will gradually expand to form cracks.

Finally, we discussed fracture behavior together with the underlying failure mechanisms in the post-necking stage, which also strongly influences the fracture resistance of such alloys for their structural applications. Figure 8 shows the fractography images of the AC and WQ samples. Generally speaking, the phase interfaces in materials serve as preferential sites for the initiation and propagation of micro-voids due to the strain incompatibility between hard and soft phases^51^. For the AC sample, many micro-voids were observed in the fracture sub-surface, see Fig. 8a. These voids are uniformly distributed in the α_s_/β (yellow arrows) and α_p_/β (red arrows) interfaces due to relatively homogeneous plastic deformation between α and β phases (Fig. 8a). The corresponding fracture surface images show many deeper and larger dimples and a few voids, indicating that the AC Ti alloys are ductile fracture via the growth and coalescence of micro-voids, see Fig. 8b, b1. Moreover, note that in the center of the fracture surface there are some voids and small dimples, but no long cracks, see Fig. 8b2. This phenomenon is attributed to the fact that thick α_s_ plates could deform homogeneously, which in turn suppresses crack initiation and propagation for enhanced fracture resistance.

For our designed hierarchical nano-martensite Ti alloy, there are two pathways to hinder crack propagation for the observed great ductility. First, micro-voids are initially formed at sites with remarkable strain incompatibility, e.g., α'/β (yellow arrows) and α_p_/β (red arrows) PBs in WQ Ti alloys, see Fig. 8c. Furthermore, the fracture surface image indicates many smaller and shallower dimples in the WQ samples, while it contains numerous voids, see Fig. 8d, d1. These micro-voids will gradually expand to form cracks, see Fig. 8d2. Subsequently, the soft β lamellae could trap cracks or inhibit their propagation by blunting the crack tip in WQ samples^52^. Obviously, when a crack propagates at a PB, owing to the 3D α'/β network, it may either run into the soft β phase or be deflected to a different path, as marked by the yellow dash line in Fig. 8c. Therefore, compared to α_s_/β lamellae in AC samples, it appears that the α'/β nanolamellae make a greater contribution to hinder crack propagation for enhanced fracture resistance since the β-transus microstructure has higher mechanical strength and a higher density of PBs. Simultaneously, the soft α_p_ phase could not only resist cracking at PBs (as marked by red arrows in Fig. 8c), but also could alleviate local stress concentrations via storing dislocations during plastic deformation^23^. Therefore, the heterogeneous microstructure confers the present WQ Ti alloys, in addition to ultra-high strength and great ductility, excellent fracture resistance for promising engineering applications.

In this work, we propose an alloy design strategy that hierarchical nano-martensites with an average thickness of ~20 nm is introduced into a cost-effective duplex Ti alloy, utilizing the diffusion mismatch between fast diffusive Cr and slow diffusive Al to architecture a high density of CBs. We can tailor the fast-diffusive element content to control the density of CBs, which in turn restricts the martensitic phase transformation during quenching. The hierarchical nano-martensite engineering strategy can potentially be applied to many other alloys without complex thermomechanical processing and expensive doping elements, including advanced steels and multicomponent alloys, especially the ability to form quenched martensites in a metastable matrix.

## Methods

### Alloy fabrication

The designed Ti-2.8Cr-4.5Zr-5.2Al alloy in weight percentage (wt.%) was casted by vacuum arc melting. A 20 kg ingot was melted from industrial purity Ti sponges, Cr granules, a high purity Zr plate and Al shots. The actual chemical compositions of the ingot were measured to be 2.82% Cr, 4.48% Zr, 5.26%Al and the balance Ti (wt.%) with unavoidable elements such as O (0.12%), N (0.014%), C (0.01%) and H (0.001%) by inductively coupled plasma-atomic emission spectrometry (ICP-AES). The β-transus temperature (T_β_) of the alloy was determined to be ~955 °C by the metallographic method.

The as-casted ingot was homogenized at 1050 °C for 12 h under vacuum, followed by β-forging at 1050 °C to obtain 100 mm thick slab. Subsequently, the slab was forged by a through-transus-processed forging starting at ~980 °C and ending at ~700 °C to produce 30 mm thick plates with ~70% thickness reduction. The forged plates were solution treated in the (α + β) phase region at 920 °C for 1 h with two types of cooling conditions, i.e., air cooling and water quenching (referred to as AC samples and WQ samples, respectively).

### Mechanical properties test

Flat dog-bone-shaped tensile specimens with a thickness of 2 mm and a gauge section of 15 × 3.5 mm^2^ were machined from the center region of heat-treated plates along the forging direction (RD) to avoid the surface effect on the microstructure. Uniaxial tensile tests were performed on an Instron 5969 universal testing machine at room temperature, with an initial strain rate of 1 × 10^−3^ s^−1^. Five specimens were tested to ensure the repeatability of mechanical testing. Tensile properties, e.g., yield strength (*σ*_y_), ultimate tensile strength (*σ*_*UTS*_) and elongation to fracture (*ε*_*f*_) were recorded. The hardness of the α_p_ grains and α'/β regions was measured using a TI950 TriboIndenter (Hysitron, Minneapolis, MN) with a standard Berkovich tip at room temperature. The hardness test was conducted on the load-controlled mode with the prescribed loading of ~3000 μN under the loading time of 5 s, correspondingly the loading strain rates of ~0.1 s^−1^. The holding time is 2 s, and the unloading time is 5 s. In the present study, the allowable-drift-rate was set at 0.02 nm s^−1^, which is 5-fold smaller than the typical value (0.1 nm s^−1^) generally used in typical nanoindentation compression tests for improve the reliability and accuracy of the measurements.

### Microstructural characterization

Microstructural observations were performed using Olympus PMG3 optical microscopy (OM), JEOL JSM-6700F scanning electron microscopy (SEM) and electron backscatter diffraction (EBSD) embedded in the SEM microscope, respectively. The specimens were mechanically polished, followed by electro-polishing for 10–20 s, and etching was performed in a solution mixture of 2:8:90 of HF, HNO_3_, and H_2_O in volume fractions. The phase constitution of the specimens was examined with a Bruker D8 Discover powder X-ray diffractometer (XRD) using a Cu K_α_ radiation (*λ* = 1.5406 Å) source at an acceleration voltage of 40 kV and a current of 100 mA. A transmission electron microscope (TEM, JEM-2100) operated at 200 kV was employed to reveal the microstructural features. The chemical distributions of microstructural constituents were analyzed by energy dispersive X-ray spectrometer (EDS). Needle-shaped specimens required for APT were fabricated by lift-outs and annular milled in an FEI Scios focused ion beam/scanning electron microscope (FIB/SEM). The APT experiments were performed on a CAMECA local electrode atom probe (LEAP 4000XSi) under a high vacuum (Pa) at 20 K. Then, using the Integrated Visualization & Analysis Software (IVAS) version 3.6.8 for the three-dimensional reconstructions and compositional analyses of the APT data of the specimens.

### Phase field modeling

Since the obvious inhomogeneity of the β-stabilizer Cr can be observed experimentally in Ti-Cr-Zr-Al alloys, a binary system Ti-2.8Cr is selected to describe Ti-2.8Cr-4.5Zr-5.2Al in our simulations for the sake of simplicity. The chemical free energy curves of the α phase and β phase can be obtained through the Pandat thermodynamic database, see Supplementary Table 3. Accordingly, the free energy expressions can be fitted by a polynomial, and the coefficients are linear with temperature. More details can be found in the Supplementary Note 5.

## Supplementary information

Supplementary Information

## Data Availability

The data that support the findings of this study are available from the corresponding author upon request.
