# Supplementary material for: Hierarchical nano-martensite-engineered a low-cost ultra-strong and ductile titanium alloy
Source: Nat Commun. 2022 Oct 10;13:5966. doi: 10.1038/s41467-022-33710-1 (PMC9550820; doi:10.1038/s41467-022-33710-1)
Supplement: Supplementary file 1 — Supplementary Information [file 41467_2022_33710_MOESM1_ESM.pdf]

Supplementary information for

Hierarchical nano-martensite-engineered a low-cost ultra-strong and ductile titanium alloy

Chongle Zhang[1,§], Xiangyun Bao[1,§], Mengyuan Hao[2,§], Wei Chen[1,§], Dongdong Zhang[1], Dong Wang[1, 2], Jinyu Zhang[1,*], Gang Liu[1], Jun Sun[1,*]

1. State Key Laboratory for Mechanical Behavior of Materials, Xi'an Jiaotong University, Xi'an 710049, People's Republic of China

2. Center of Microstructure Science, Frontier Institute of Science and Technology, Xi'an Jiaotong University, Xi'an 710049, China

§. These authors contribute equally to this work.

*E-mail address: jinyuzhang1002@xjtu.edu.cn (Jinyu Zhang)

junsun@mail.xjtu.edu.cn (Jun Sun)

Content:

Supplementary Figure 1. Microstructural characterization of the Ti-xCr-4.5Zr-5.2Al alloys.
Supplementary Figure 2. Mechanical behaviors of the Ti-xCr-4.5Zr-5.2Al alloys.
Supplementary Figure 3. Schematic illustration of the thermomechanical processing route performed in the present Ti-xCr-4.5Zr-5.2Al alloys.
Supplementary Figure 4. Microstructure characterization in WQ Ti alloys.
Supplementary Figure 5. XRD profiles of the present AC and WQ Ti-Al-Cr-Zr alloys.
Supplementary Figure 6. The microstructure and distribution of alloying elements in our β-transus forged Ti alloys.
Supplementary Figure 7. The chemical free energy of the α and β phases at different temperature.
Supplementary Figure 8. Calculated α precipitate morphology and concentration distribution after different cooling rates.
Supplementary Figure 9. Nanoindentation measurements for the WQ Ti alloy.
Supplementary Figure 10. EBSD analysis of the different phases in present WQ and AC alloys.
Supplementary Figure 11. EBSD analysis of the α_p phases in present WQ alloy
Supplementary Figure 12. A comparison of the theoretical yield strength and measured yield strength σ_y for the present AC and WQ Ti alloys.
Supplementary Table 1. Phase composition of the present WQ, AC and β-transus forged Ti alloys.
Supplementary Table 2. The average metal cost of alloying elements.
Supplementary Table 3. The basic parameters of the present phase field simulations.
Supplementary Table 4. The physical constants used for strength calculation for AC and WQ Ti alloys.
Supplementary Note 1. Composition design to prepare nano-martensite Ti alloys.
Supplementary Note 2. Thermomechanical processes to prepare hierarchical nano-martensite Ti-Cr-Zr-Al alloys.
Supplementary Note 3. The phases and crystallographic orientations in AC and WQ Ti-Cr-Zr-Al alloys.
Supplementary Note 4. Detailed composition, microstructural and cost analysis for the present hierarchical Ti alloys.
Supplementary Note 5. Phase field modeling.
Supplementary Note 6. The nanoindentation test for the strength discrepancy between α_p and α′/β phases.
Supplementary Note 7. A comparison of the theoretical yield strength and measured yield strength.
Supplementary References

Supplementary Note 1. Composition design to prepare nano-martensite Ti alloys

The chemical boundaries (CBs) is defined as a sharp discontinuity of at least one elemental concentration inside a lattice-continuous region[1]. Apparently, these CBs (corresponds to the concentration fluctuations) are created by the diffusion mismatch of alloying elements in a β grain before phase transformation at high temperature. The density of CBs before the phase transformation was controlled by the diffusion length of alloying elements. Therefore, tailoring the Cr content (fast diffusing element) in the current alloy system means regulating the degree of chemical fluctuation during quenching, i.e., the density of GBs.

Based on above conceptual design and simulations, we in this work for the first-time architecture nano-martensites (with thickness only ~20 nm, far less than 50 nm) via CBs to significant enhance the mechanical properties of low-cost Ti alloys. Because the chemical discontinuity spacing is governed by the diffusion length of alloying elements[2], fast diffusing elements contribute to the formation of larger composition fluctuations or a higher density CBs during rapid cooling. Subsequently, we tailor the density of CBs at high temperature by tuning the Cr concentrations, thereby regulating the subsequent phase transformation behavior. To verify our design strategy and obtain much refined nano-martensites, we fabricated three Ti-*x*Cr-4.5Zr-5.2Al (x = 1.8, 2.3 and 2.8 wt.%) alloys with different Cr (the β stability element) contents. Moreover, to obtain similar microstructures, three alloys were processed and heat-treated in the same way based on the β-transus temperature. For example, all these Ti-*x*Cr-4.5Zr-5.2Al samples underwent solution treatment in the (α+β) phase field (~30 °C below the β-

transsus temperature, see Supplementary Fig. 3) and showed quenched martensitic structure, see Supplementary Fig. 1a1-c1. It is found that the martensite thickness is reduced from ~119 to ~20 nm as the Cr content is increased from 1.8 to 2.8 wt.%, see Supplementary Fig. 1a2-c2. In other words, the methodology to control the size of CBs was successfully realized by tailoring the Cr concentration. Obviously, this is a record fine α′-martensitic lamellar thickness (~20 ± 6 nm) among all martensite Ti alloys reported to date when the Cr content is 2.8 wt.%, see Fig. 3h.

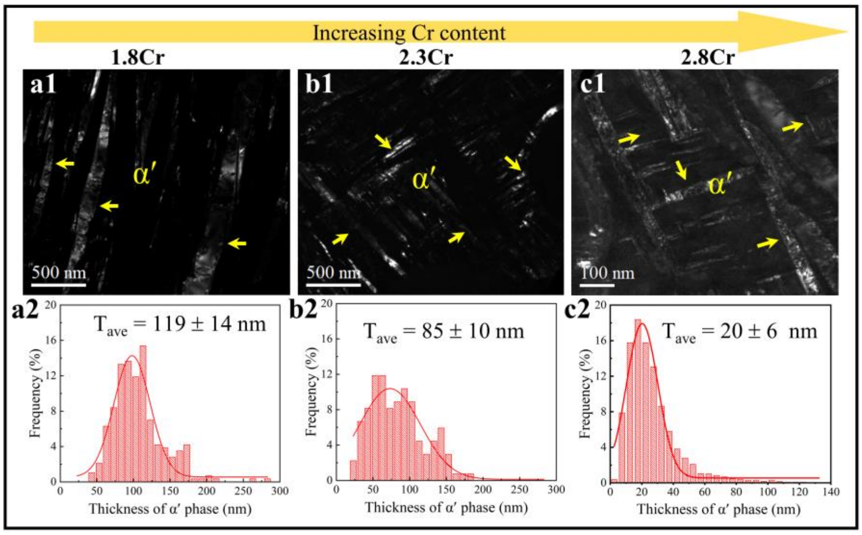

Supplementary Figure 1. Microstructural characterization of the Ti-*x*Cr-4.5Zr-5.2Al alloys. **a1**, **b1** and **c1** Dark-field transmission electron microscopy (TEM) images showing the martensite α′ phase in three Ti-*x*Cr-4.5Zr-5.2Al alloys. **a2**, **b2** and **c2** The statistical distribution of the thicknesses of martensite. **a1** and **a2** correspond to Ti-1.8Cr-4.5Zr-5.2Al alloy; **b1** and **b2** Ti-2.3Cr-4.5Zr-5.2Al alloy; **c1** and **c2** Ti-2.8Cr-4.5Zr-5.2Al alloy.

The representative engineering stress–strain curves of the water quenched Ti-*x*Cr-4.5Zr-5.2Al alloys are shown in Supplementary Fig. 2. It appears that because the martensite thickness decreases with increasing the Cr content (see Supplementary Fig.

1), the yield strength of our designed Ti alloys is significantly improved, while their ductility is almost constant. Compared with 1.8Cr samples, the yield strength of 2.8Cr alloys is increased by about 393 MPa. Although Cr doping can induce solid solution strengthening, the contribution of 1 wt.% Cr to the strengthening is only ~31 MPa[3,4]. Obviously, the contribution of yield strength origin from the notable increase of α′/β interface density, and the strengthening contribution is ~362 MPa. Therefore, we only focus on the microstructure and mechanical properties of Ti-2.8Cr-4.5Zr-5.2Al alloys to convey our alloy design strategy, which is applicable to other metastable alloys.

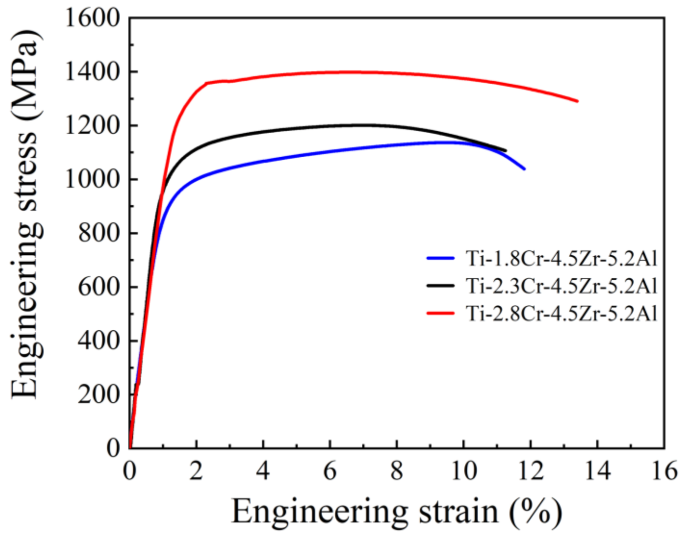

Supplementary Figure 2. Mechanical responses of our designed Ti alloys with different Cr contents. Tensile engineering stress-strain curves of Ti-*x*Cr-4.5Zr-5.2Al (x = 1.8, 2.3 and 2.8 wt.%) alloys at room temperature.

Supplementary Note 2. Thermomechanical processes to prepare nano-martensite Ti alloys

In this study, we employed the multiple through-transus-processed forging to refine β-grain size of Ti alloys, as shown in Supplementary Fig. 3.

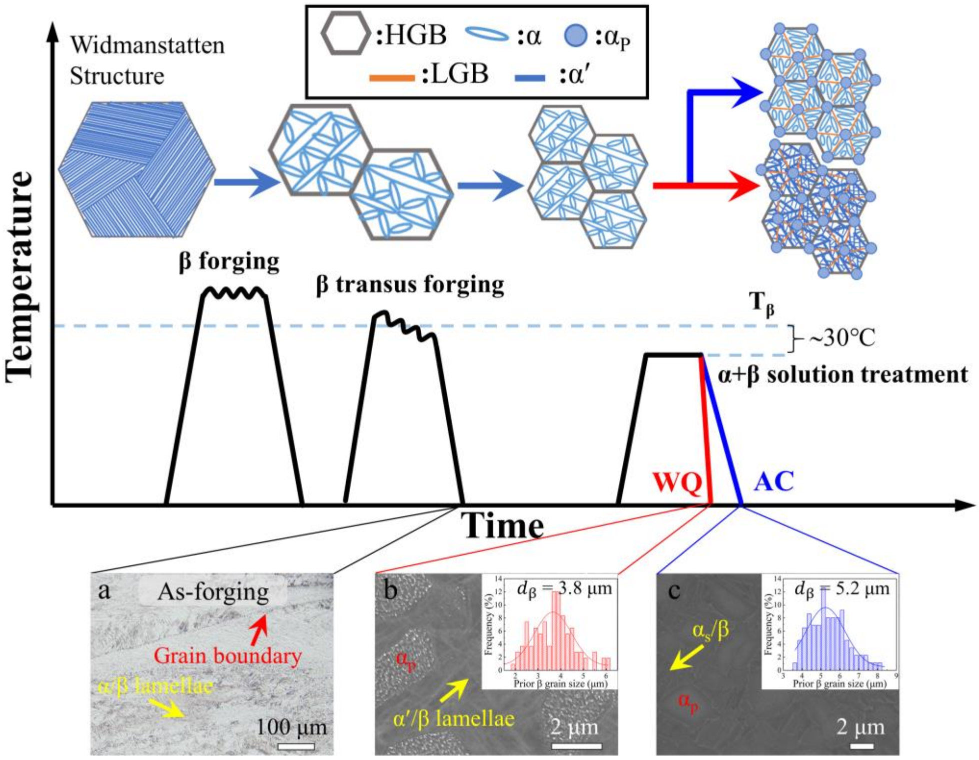

Supplementary Figure 3. Schematic illustration of the thermomechanical processing route performed in the present Ti-*x*Cr-4.5Zr-5.2Al alloys. (HGB: High-angle grain boundary, LGB: Low-angle grain boundary). Optical micrographs of the Ti-2.8Cr-4.5Zr-2.5Al alloys under different states. **a** as-forged, **b** WQ and **c** AC (see Supplementary Fig. 3 for processing details). The insets in **b** and **c** are statistical grain size distributions of the prior β grains in WQ and AC samples, respectively.

Supplementary Note 3. The phases and crystallographic orientations in AC and WQ Ti alloys.

TEM and XRD tests were used to measure lattice parameter, see Supplementary Fig. 4-5. The diffraction pattern (Supplementary Fig. 4b) was indexed to be HCP structure, corresponding to α′ phase with d-spacings determined for the $\{10\bar{1}0\}$ planes (2.54 Å, see Supplementary Fig. 4c), which was consistent with the previously observed value (2.54 - 2.61 Å)[5]. On the other hand, XRD analysis of the WQ sample has shown there is the α′-martensite phase with the HCP crystal structure and lattice parameters *a* = 2.932 Å and *c* = 4.669 Å, see Supplementary Fig. 5. These lattice parameters are similar to those reported by Welch et al (*a* = 2.931 Å; *c* = 4.681 Å)[6] and Kaschel et al (*a* = 2.933 Å; *c* = 4.655 Å)[5]. Therefore, the microstructure produced is martensitic α′.

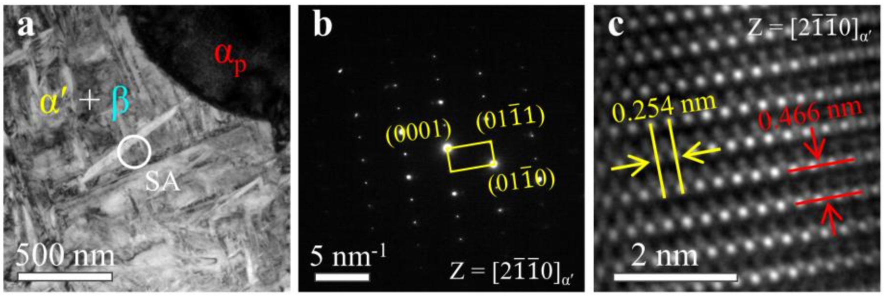

Supplementary Figure 4. Microstructure characterization in WQ Ti alloys. a The bright field (BF) transmission electron microscopy (TEM) image showing the primary α precipitates and the transformed microstructure in a β-grain. **b** The corresponding corresponding selected area electron diffraction (SAED) pattern of the circle region in **a**. **c** The high-resolution TEM image showing the lattice constant of the α′ phase.

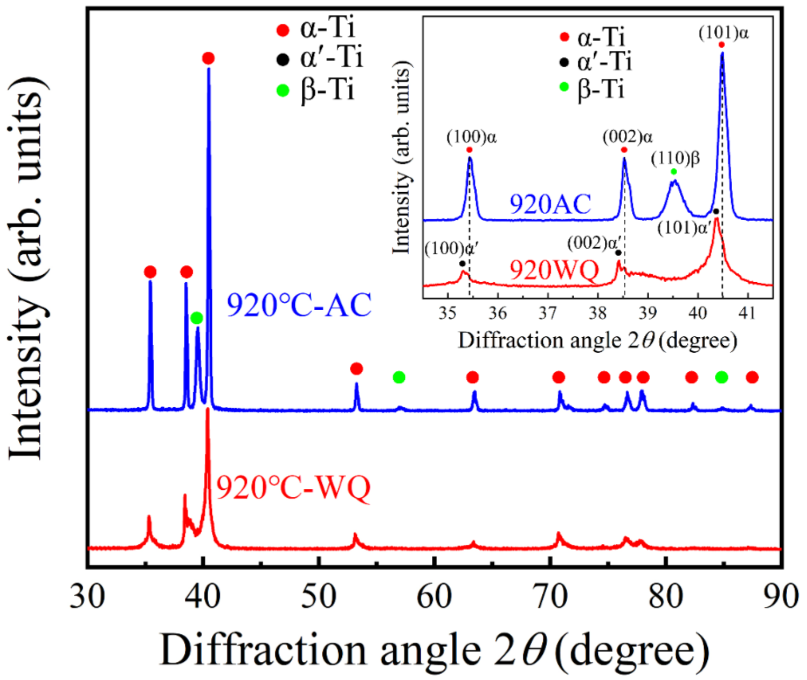

Supplementary Figure 5. XRD profiles of the present AC and WQ Ti alloys. XRD spectra showing that the WQ sample contains α, α′, and β phases, whereas the AC sample has α and β phases.

Supplementary Note 4. The composition and cost of the present β-transsus forged Ti alloys.

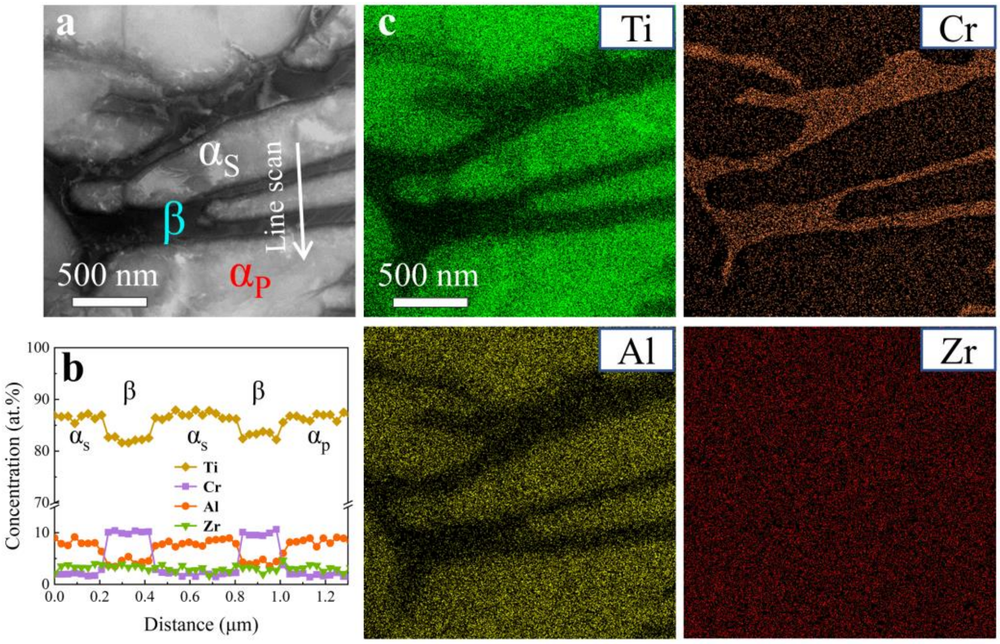

Supplementary Figure 6. The microstructure and distribution of alloying elements in β-transus forged Ti alloys. a A high-angle annular dark-field scanning TEM (HAADF-STEM) image. **b-c** The corresponding EDS line and mapping analysis in **a**.

Supplementary Table 1. Phase composition (in at. %) of the present WQ, AC and β-transus forged Ti alloys.

	Phase type	Ti	Cr	Zr	Al
WQ	β	82.85 ± 0.41	6.83 ± 0.32	3.52 ± 0.21	6.32 ± 0.26
	α_p	86.94 ± 0.39	0.95 ± 0.17	2.92 ± 0.29	8.92 ± 0.43
	α′	87.75 ± 0.52	1.11 ± 0.24	2.89 ± 0.31	8.21 ± 0.47
AC	β	82.61 ± 0.63	10.86 ± 0.78	3.81 ± 0.37	4.63 ± 0.41
	α_p	87.50 ± 0.29	0.98 ± 0.23	2.87 ± 0.38	8.63 ± 0.44
	α_s	87.66 ± 0.72	0.83 ± 0.17	3.13 ± 0.48	8.37 ± 0.53
β-transus forged	β	82.22 ± 0.43	10.32 ± 0.21	3.40 ± 0.32	4.27 ± 0.59
	α_p	87.14 ± 0.56	0.75 ± 0.23	2.82 ± 0.45	9.23 ± 0.55
	α_s	87.01 ± 0.72	0.97 ± 0.21	2.89 ± 0.55	8.19 ± 0.52

Note: Phase composition of WQ sample measured from APT analysis and TEM-EDS. The errors are determined based on EDS counting statistics.

The alloy cost was calculated using the estimated cost by alloy addition found in ref.[7,8]. Those values fluctuate considerably according to demand and they are used here only for the sake of relative comparison.

Supplementary Table 2. The average metal cost of alloying elements[7,8].

Element	Price (USD kg^{-1})	Role in phase stability
Ti	11.7	
Cr	15.4	β-stability
Mo	44.5	β-stability
V	396.9	β-stability
Al	2.7	α-stability
Zr	35	Neutral
Sn	20.4	Neutral

Supplementary Note 5. Phase field modeling

Since the obvious inhomogeneity of β stabilized element Cr can be observed experimentally in Ti-Cr-Zr-Al alloys, to simplify the simulations, a binary system Ti-2.8Cr is selected to describe Ti-2.8Cr-4.5Zr-5.2Al in our simulations. The chemical free energy curves of α phase and β phase can be obtained through Pandat thermodynamic database as shown in Supplementary Fig. 7. Accordingly, the free energy expressions can be fitted by a polynomial, and the coefficients are linear with temperature.

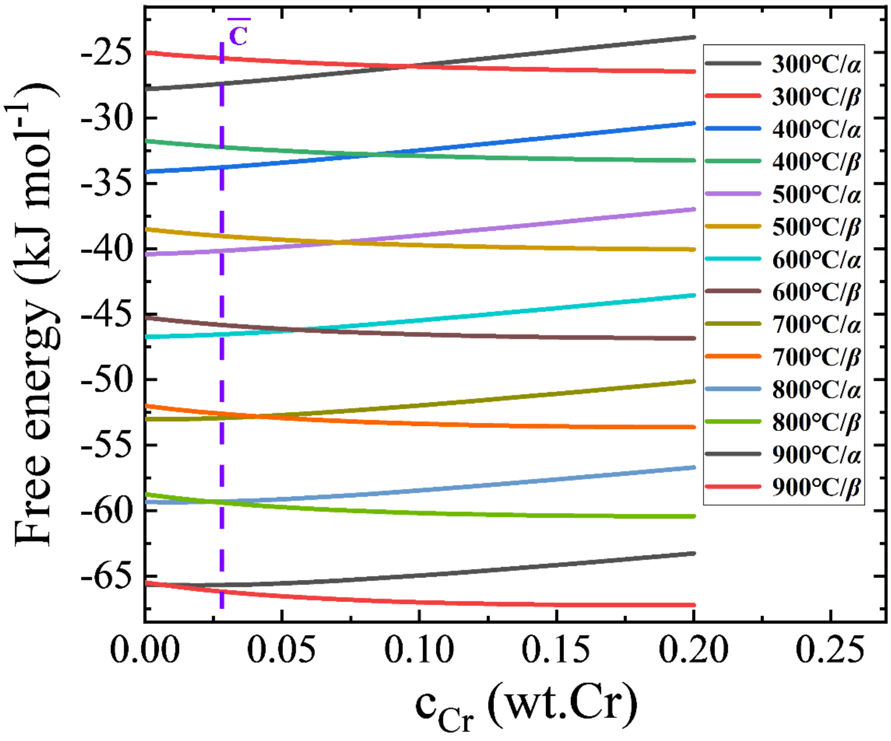

Supplementary Figure 7. The chemical free energy of the α and β phases at different temperature. Chemical-free energy curves of the α and β phases for Ti-Cr systems at different temperatures (300 °C to 900 °C with the 100 °C interval). The purple dotted line marks the initial average composition $\bar{c}$ = 2.8%. The unit of composition is mass percentage.

The total free energy can be expressed by non-conserved structure field (η) and conserved structure field (c)[9-11]:

$$F = \int_V [\sum_p h(\eta_P) g_\alpha(c_{Cr}, T) + (1 - \sum_P h(\eta_P)) g_\beta(c_{Cr}, T) + \omega_1 \sum_P \sum_{p \neq q} \eta_P \eta_q + \sum_p \omega_2 (\eta_P^2 - 2\eta_P^3 + \eta_P^4) + \frac{k}{2} (\nabla c_{Cr})^2 + \frac{\varepsilon}{2} \sum_P (\nabla \eta_P)^2 + E^{elastic} \quad (1)$$

where c_{cr} stands for the concentration of chromium, η_p stands for the structure field of the p_{th} α variants. w_1 and w_2 are the energy barrier between the α phase and β phase and among different α variants, respectively. ε and κ represent the gradient energy coefficients of the structure field and concentration field, respectively[9]. $g_\alpha(c_{Cr}, T)$ and $g_\beta(c_{Cr}, T)$ describe the composition and temperature dependence of chemical free energy of α and β phases from Pandat database as follows:

$$g_\alpha(c_{Cr}, T) = (-63.1T - 8873.7) + (-32.2T + 21744.9)c_{Cr} + (211.7T + 43577.6)c_{Cr}^2 + (-1098.6T - 303505.5)_{Cr}^3 + (3809.3T + 1.03975E6)c_{Cr}^4 + (-8013.88T - 2.187E6)c_{Cr}^5 + (9924.7T + 2.70651E6)c_{Cr}^6 + (-6638.92T - 1.8094E6)c_{Cr}^7 + (1850.98T + 504229)c_{Cr}^8 \quad (2)$$

and

$$g_\beta(c_{Cr}, T) = (-67.5T - 4757.01646) + (-19.9T - 12819.8)c_{Cr} + (205.1T + 63819.9)c_{Cr}^2 + (-1098.2T - 300949.4)c_{Cr}^3 + (3801.8T + 1.04536E6)c_{Cr}^4 + (-7995.2T - 2.1997E6)c_{Cr}^5 + (9898.86T + 2.72411E6)c_{Cr}^6 + (-6620.5T - 1.82194E6)c_{Cr}^7 + (1845.62T + 507869)c_{Cr}^8 \quad (3)$$

This simple interpolation function $h(\eta)$ is commonly used to connect two free energy curves as follows:

$$h(\eta) = \eta^3(6\eta^2 - 15\eta + 10) \quad (4)$$

When the total free energy is minimized with respect to the structure field, we can obtain two solutions. One is $h = 1$, which corresponds to the equilibrium free energy

curve of phase α, and the other is $h = 0$, corresponding to the equilibrium free energy curve of phase β.

The contribution of elastic interaction energy can be derived according to the khachaturyan-shatalov theory as follows[12]:

$$E^{elastic} = \frac{1}{2}\int_V \frac{d\vec{k}}{2\pi^3} \sum_{p,q}^{3} B_{pq}(\vec{n})\tilde{\eta}_P(\vec{k})\tilde{\eta}_q^*(\vec{k}) \quad (5)$$

in which B_{pq} is the transformation strain function for each α variant as follows:

$$B_{pq}(\vec{n}) = C_{ijkl}\varepsilon_{kl}^T(p)\varepsilon_{kl}^T(q) - \vec{n}\sigma_{ij}^T(p)\Omega_{ij}(\vec{n})\sigma_{kl}^T(q)\vec{n}_l \quad (6)$$

where C_{ijkl} stands for the stiffness tensor, stress-free transformation strain of the pth variant is represented by $\varepsilon_{ij}^T(p)$, and $\sigma_{ij}^T(p) = C_{ijkl}\varepsilon_{kl}^T$, $[\Omega(\vec{n})]_{ik}^{-1} = C_{ijkl}n_j n_l[\Omega(\vec{n})]$. It should be noted that coherent boundary assumption is used in our simulations because our work focuses on the early stage of α nucleation[9].

In our simulations, three equivalent α-variants with 120° rotation are shown in the $(11\bar{1})$ plane which has 512 × 512 nm computational units, and the stress-free transformation strain can be calculated based on Burgers orientation and lattice constant $a_\beta = 3.254$ Å, $a_\alpha = 2.945$ Å, $c_\alpha = 4.674$ Å.

By solving the Chan-Hilliard (CH) equation and Ginzburg-landau (TDGL) equation for concentration field and structure field respectively, the evolution of concentration diffusion and structural transformation can be obtained[13]:

$$\frac{\partial c_{Cr}}{\partial t} = \nabla(M(T)\nabla\frac{\delta F}{\delta c_{Cr}}) + \xi_c(T) \quad (7)$$

$$\frac{\partial \eta_p}{\partial t} = -L\frac{\delta F}{\delta \eta_p} + \xi_\eta(T) \quad (8)$$

where M represents the chemical mobility of the concentration field, L stands for the structural mobility assumed to be independent of temperature. ξ_c as well as ξ_η represent

the Langevin force terms of the concentration field and structural field to simulate thermal fluctuation that is closely related to temperature.

Supplementary Table 3. The basic parameters of the present phase field simulations

Parameter	Value	Ref.
nx, ny	512	
nz	1	
dx, dy, dz	1	
dt	0.1	
L	10	
$D_{Cr}(T)$	$7.6E$-11exp(-56682.7/RT) (323K<T<1173K)	14
C_{11}	160.5E9	10
C_{12}	125.6E9	10
C_{44}	34.1E9	10
w_1	0.06	
w_2	0.01	
k	60.0	
ε	0.05	
$Enorm(T)$	-400T+539200 (323K<T<1173K)	
V_m	1.0E-5	

The basic parameters used in the phase field simulations are listed in Supplementary Table 3. The dimensionless coefficients are obtained by normalized free energy factor which depends on temperature upon cooling: $E_{norm}(T) = -400T + 539200$ $(323K<T<1173K)$. By assuming a coherent interfacial energy ~0.1 J m^{-2} between β and α phases[11], the grid size with $dx = dy = dz = 1$ nm have been used in our simulations[15]. The coefficient M in phase field model represents the chemical mobility of the concentration field, which is proportional to interdiffusion coefficient D and temperature[16]: $M = D/RT$. Based on the relationship between chemical mobility and diffusion coefficient, the dimensionless time step $\triangle t$ ~1.0 s in our simulations.

The cooling rate is determined by the magnitude of the temperature change per

unit of simulation time. The faster the cooling rate, the greater the temperature change per unit of simulation time, and vice versa.

Supplementary Fig. 8 shows various calculated microstructures with the increase of cooling rate from 1 °C s^{-1} to 250 °C s^{-1}, which demonstrate the size and types of α and β are closely related to cooling rate. As seen from the Supplementary Fig. 8a1, b1, the structural transformation is accompanied by a concentration field that has reached equilibrium for the slow cooling rate. There is sufficient time to homogenize the distribution of solutes associated with the reduction in chemical fluctuations with decreasing temperature, see Supplementary Fig. 8a, b. With the increase of cooling rate to V2 (50 °C s^{-1}, water quenching), the intense concentration fluctuations existed at 920 °C are still maintained at 650 °C (see Supplementary Fig. 8c, d), and fine martensite α′ are generated results from the structural transformation (see Supplementary Fig. 8c3, d3). Obviously, the concentration heterogeneity will be freezed at relatively low temperature due to the insufficient diffusion time and the fast cooling rate. The number of α′ precipitates decreases with the increase of cooling rate in this stage, see Supplementary Fig. 8e-h. It should be noted that our simulation focused on intragranular microstructure and no consideration of grain boundaries or other defects.

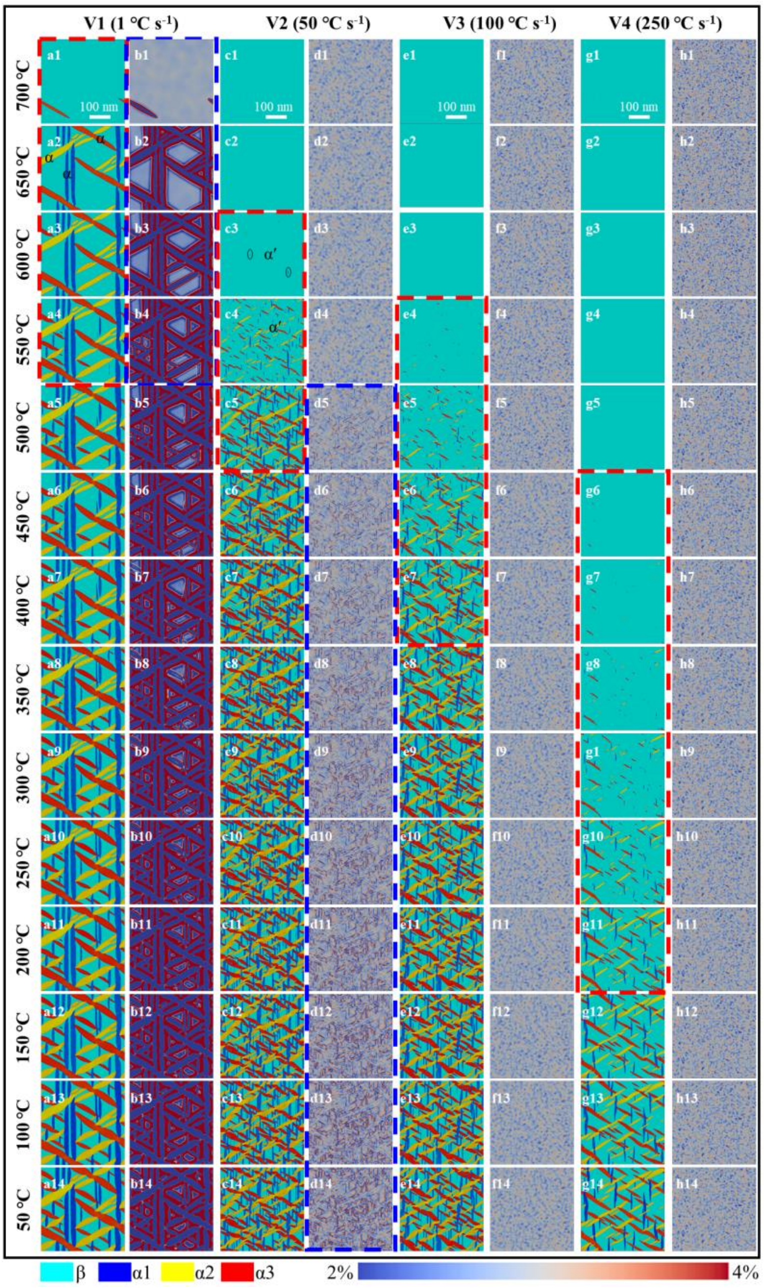

Supplementary Figure 8. Calculated α precipitate morphology and concentration distribution after different cooling rates. a1-a14, **c1-c14**, **e1-e14**, **g1-g14** The corresponding structure fields after different cooling rates. Light blue color represents the β phase, and dark blue, yellow, and red colors describe three variants of the α precipitates. **b1-b14, d1-d14, f1-f14, h1-h14** The composition fields after different cooling rates, light blue and dark blue stand for the Cr-depleted and Cr-enriched domains, respectively. The color bar refers to the Cr concentration (wt.%) and different colors distinguish the Cr-depleted domains (light blue) and Cr-enriched domains (near red).

Supplementary Note 6. The nanoindentation test for the strength discrepancy between α_p and α'/β microstructure

The hardness of the α_p grains and α'/β regions was measured using a TI950 TriboIndenter (Hysitron, Minneapolis, MN) with a standard Berkovich tip at room temperature, following the Oliver-Pharr method adopted in our previous work[17-19]. It is found that the hardness of soft α_p is ~4.8 GPa, while that of the hard α'/β microstructure is ~5.6 GPa.

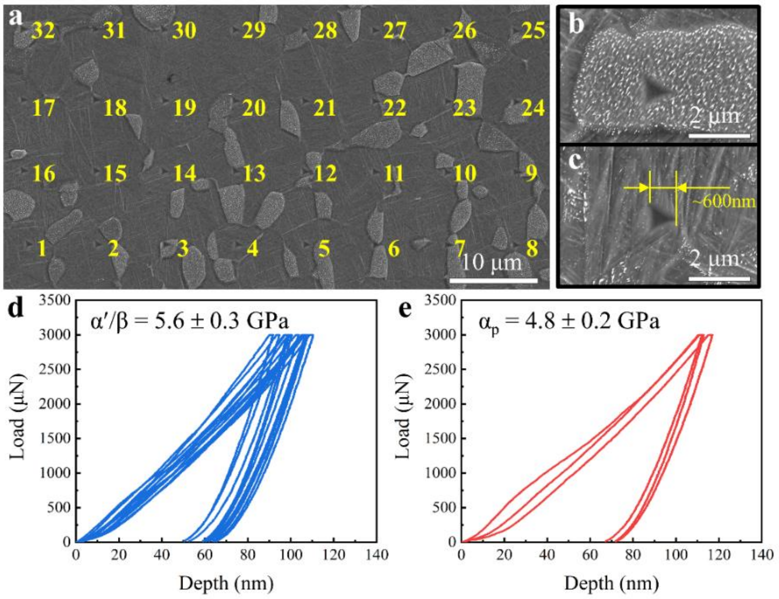

Supplementary Figure 9. Nanoindentation measurement for the WQ Ti alloy. a An SEM image showing the region of interest with 8 × 4 indent grid. **b-c** The morphologies of indentation located in the α_p phase and α'/β lamellae are displayed in **b** and **c**, respectively. **d-e** The load–displacement curves of α'/β lamellae and α_p precipitates, respectively.

Supplementary Note 7. A comparison of the theoretical yield strength and measured yield strength

According to previous studies[20-24], the yield strength for α and β_{trans} microstructure (including α′/β or α_s/β for WQ or AC samples, respectively) can be obtained via the rule of mixture, and the strength formulation then takes the form:

$$\sigma_\alpha = \sigma_{0\alpha} + \sigma_{\rho\alpha} + \sigma_{GB\alpha} \quad (9)$$

$$\sigma_\beta = \sigma_{0\beta} + \sigma_{\rho\beta} + \sigma_{GB\beta} + \sigma_{PB\beta} \quad (10)$$

further, the yield strength of alloys is expressed as:

$$\sigma_y = f_\alpha \sigma_\alpha + f_\beta \sigma_\beta \quad (11)$$

where σ_α and σ_β are the stresses of α and β_{trans} microstructure, respectively, and f_α and f_β are the corresponding volume fractions of two groups of microstructures, as shown in Supplementary Table 4.

The lattice friction stress σ_0 of a multi-element Ti alloy can be calculated by the following equation[3,24]:

$$\sigma_0 = \sigma_{Ti} + (\textstyle\sum_i B_i^{3/2} X_i)^{2/3} \quad (12)$$

Where σ_{Ti} is the lattice friction stress of pure Ti, B_i is the strengthening coefficient for solute i and X_i is the atomic percentage of alloying elements.

The forest dislocation hardening, σ_ρ, is described via the Taylor model[25]:

$$\sigma_\rho = \alpha M \mu b \sqrt{\rho} \quad (13)$$

where α is dislocation interaction constant, μ is the shear modulus, M is the Taylor factor, b is the Burgers vector (refer to Ref.[3,26] for all parameters), and ρ is the dislocation densities. The density of geometrically necessary dislocations (GNDs) was obtained

from the average KAM analysis for the present alloys, see Supplementary Table 4. For the AC sample, the dislocation density ρ was calculated based on the EBSD results (see Supplementary Fig. 10) using the following formula[27,28]:

$$\rho = \frac{2\theta_{KAM}}{Xb} \tag{14}$$

where X is the step size. θ_{KAM} is the average KAM values of the specimen, given by:

$$\theta_{KAM} = \Sigma f_i \, \theta_i \qquad \theta < 5° \tag{15}$$

where θ_i and f_i are the local misorientation and its frequency, respectively.

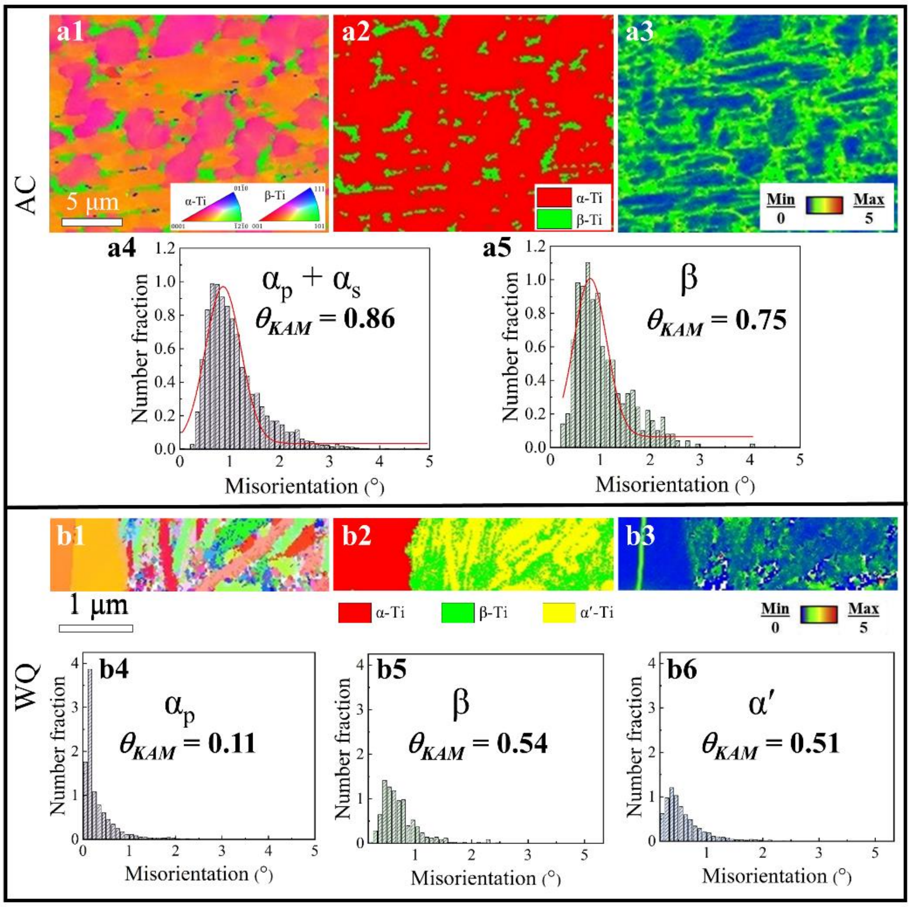

Supplementary Figure 10. EBSD analysis of the different phases in present WQ and AC alloys. a1-b1 The IPF maps. **a2-b2** The EBSD phase-map. **a3-b3** The local misorientation maps (i.e., the kernel average misorientation (KAM) maps). **a4-a5**, **b4-b6** The bar charts showing the statistical distribution of the local misorientation. **a1-a5** correspond to AC alloys; **b1-b6** correspond to WQ alloys. A step size of 120 nm and 15 nm was used during EBSD scan for AC and WQ samples, respectively.

Here, the corresponding GNDs of α′ and β lamellae in the WQ sample can be calculated as 4.2×10^{15} m^{-2} and 4.0×10^{15} m^{-2}, respectively. These values are very close to the dislocation density in the martensite of Ti alloys (about 3.9×10^{15} m^{-2})[29]. In addition, a 50 nm step size was used to identify the grain orientations of α_p grains and the step size (15 nm *vs*. 50 nm) does not affect the dislocation density of α_p grains. Obviously, Supplementary Fig. 10-11 show almost the same dislocation density for α_p grains, and the statistical results show that the dislocation densities of α_p grains with different orientations are almost the same, see Supplementary Fig. 11.

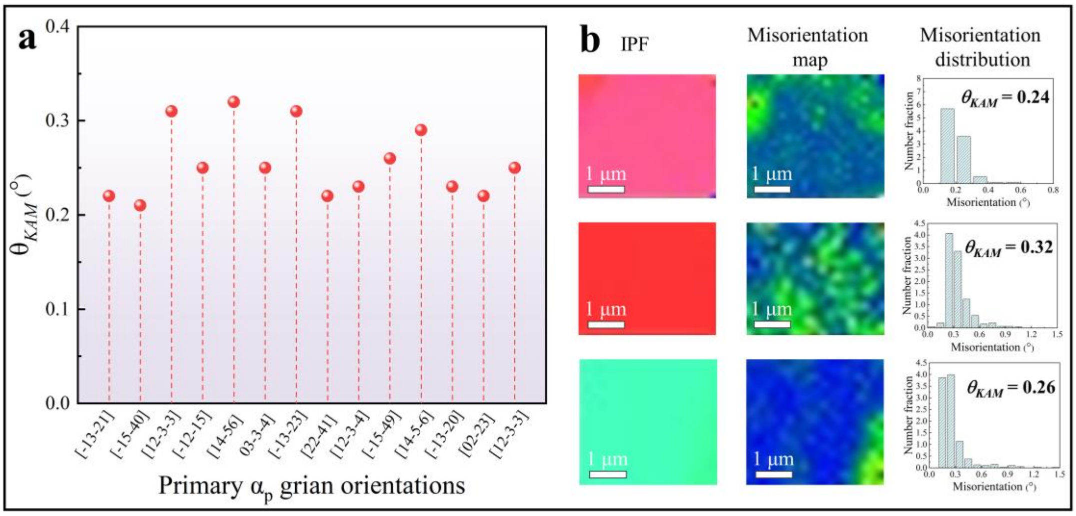

Supplementary Figure 11. EBSD analysis of the α_p phases in present WQ alloy. a The primary α_p grain with different orientations and their corresponding average KAM values. **b** Three α_p precipitates with different orientations are shown as examples. A step size of 50 nm was used during EBSD scan for the WQ sample.

The GBs strengthening can be described via the Hall-Petch relationship in polycrystalline metals[30]:

$$\sigma_{GB} = k_y d^{-1/2} \tag{16}$$

where d is the grain size (including prior β grain size and α_p particle size), and k_y is the H-P slope[20,24].

It was documented that a Hall-Petch relationship also describes strengthening contribution of nano-lamellar phase boundaries[31,32]. Duo to dislocations initially start to propagate inside the soft phase and pile-up at interphase boundaries, the strength is determined by the thickness and properties of the soft phase. In the nano-martensitic duplex architecture, compare to α′ lamellae, BCC β lamellae are the soft phase[33]. Here, the strength contributed by the α′/β boundary can be evaluated by[31,32]:

$$\sigma_{PB} = k_l \lambda^{-1/2} \tag{17}$$

where k_l is the Hall–Petch coefficient and λ is the average thickness of the β lamellae. The Hall–Petch coefficient k_l of phase boundaries can be calculated by:

$$k_l = \left(\frac{n^2\mu^2 b^2}{8\lambda}\right)^{1/2} \tag{18}$$

where n = ~3 is the number of dislocations crossing the same lamellae (obtained through TEM analysis of the small strained sample, see Fig. 7b2). Using these data in Supplementary Table 4, the strengthening contribution by the nano-lamellar boundaries was estimated to be ~421 MPa, providing the main contribution to the macroscopic yield strength for WQ sample. It appears that the calculated strengths are in good agreement with experimental results of WQ and AC samples, as marked in Supplementary Fig. 12.

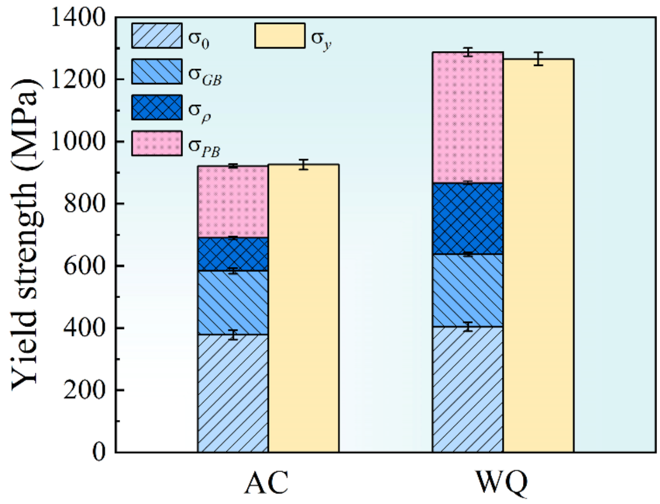

Supplementary Figure 12. A comparison of the theoretical yield strength and measured yield strength σ_y for the present AC and WQ Ti alloys. The strength contribution originates from solution strengthening, forest dislocation hardening, grain boundaries strengthening and phase interface strengthening. Error bars represent standard deviation.

Supplementary Table 4. The physical constants used for strength calculation for AC and WQ Ti alloys.

Parameter	Description	Sample type						Ref.
		WQ			AC			
		α_p	α'	β	α_p	α_s	β	
K_y	Hall-Petch constant (MPa·m$^{-1/2}$)	0.15		0.5	0.15		0.5	[20,24]
d_β	Prior β grain size	3.8 μm			5.2 μm			This work
d_α	α_p phase size	1.2 μm			2.9 μm			This work
K_l	Hall-Petch constant (PBs)	0.084 MPa ·m$^{-1/2}$			0.091 MPa ·m$^{-1/2}$			This work
λ_β	The thickness of β lamellae (nm)	20 ± 5 nm			75 ± 8 nm			This work
ρ_0	Dislocation density (10^{15} m^{-2})	8.4	40	42	8.4		7.7	This work, [29]
μ	Shear modulus (GPa)	44	44	39	44	44	39	[3,26]
a	Dislocation interaction constant	0.2	0.2	0.3	0.2	0.2	0.3	[34]
b	Burgers vector magnitude (Å)	2.95	2.95	2.8	2.95	2.95	2.8	[3,26]
M	Taylor factor	1	1	2.8	1	1	2.8	[3,34]
f	Volume fraction (%)	20	58.7	21.3	30	52.4	17.6	This work

Supplementary References

1. Ding R, et al. Chemical boundary engineering: A new route toward lean, ultrastrong yet ductile steels. *Sci Adv* **6**, eaay1430 (2020).
2. Zhang D, et al. Additive manufacturing of ultrafine-grained high-strength titanium alloys. *Nature* **576**, 91-95 (2019).
3. Zhao G-H, Xu X, Dye D, Rivera-Díaz-del-Castillo P E J. Microstructural evolution and strain-hardening in TWIP Ti alloys. *Acta Mater* **183**, 155-164 (2020).
4. Zhao G H, Liang X Z, Kim B, Rivera-Díaz-del-Castillo P E J. Modelling strengthening mechanisms in beta-type Ti alloys. *Mater Sci Eng A* **756**, 156-160 (2019).
5. Kaschel F R, et al. Mechanism of stress relaxation and phase transformation in additively manufactured Ti-6Al-4V via in situ high temperature XRD and TEM analyses. *Acta Mater* **188**, 720-732 (2020).
6. Welsch G, Boyer R, Collings E. *Materials properties handbook: titanium alloys*. ASM international (1993).
7. Abd-elrhman Y, Gepreel M A H, Abdel-Moniem A, Kobayashi S. Compatibility assessment of new V-free low-cost Ti–4.7Mo–4.5Fe alloy for some biomedical applications. *Mater Des* **97**, 445-453 (2016).
8. Fernandez V. Assessing cycles of mine production and prices of industrial metals. *Resources Policy* **63**, 101405 (2019).
9. Boyne A, et al. Pseudospinodal mechanism for fine α/β microstructures in β-Ti alloys. *Acta Materialia* **64**, 188-197 (2014).
10. Zhang T, Wang D, Wang Y. Novel transformation pathway and heterogeneous precipitate microstructure in Ti-Alloys. *Acta Materialia* **196**, 409-417 (2020).
11. Zhang T, et al. Non-conventional transformation pathways and ultrafine lamellar structures in γ-TiAl alloys. *Acta Materialia* **189**, 25-34 (2020).
12. Khachaturyan A G. *Theory of structural transformations in solids*. Courier Corporation (2013).
13. Hao M, et al. Heterogeneous Microstructure Enhanced Comprehensive Mechanical Properties in Titanium Alloys. *Jom* **73**, 3082-3091 (2021).
14. Lee S Y, Iijima Y, Hirano K. DIFFUSION OF CHROMIUM AND PALLADIUM IN BETA-TITANIUM. *Materials Transactions Jim* **32**, 451-456 (1991).
15. Shi R, Wang Y. Variant selection during α precipitation in Ti–6Al–4V under the influence of local stress – A simulation study. *Acta Mater* **61**, 6006-6024 (2013).
16. Wheeler A A, Boettinger W J, McFadden G B. PHASE-FIELD MODEL FOR ISOTHERMAL PHASE-TRANSITIONS IN BINARY-ALLOYS. *Physical Review A* **45**, 7424-7439 (1992).
17. Niu J J, et al. Size-dependent deformation mechanisms and strain-rate sensitivity in nanostructured Cu/X (X=Cr, Zr) multilayer films. *Acta Mater* **60**, 3677-3689 (2012).
18. Zhao J T, et al. Zr alloying effect on the microstructure evolution and plastic deformation of nanostructured Cu thin films. *Acta Mater* **132**, 550-564 (2017).
19. Zhang J Y, et al. Alloying effects on the microstructure and mechanical properties of nanocrystalline Cu-based alloyed thin films: Miscible Cu-Ti vs immiscible Cu-Mo. *Acta Mater* **143**, 55-66 (2018).
20. Tan C, Sun Q, Xiao L, Zhao Y, Sun J. Slip transmission behavior across α/β interface and strength prediction with a modified rule of mixtures in TC21 titanium alloy. *J Alloys Compd* **724**, 112-120 (2017).

21. Huang C, et al. Effect of microstructure on tensile properties of Ti–5Al–5Mo–5V–3Cr–1Zr alloy. *J Alloys Compd* **693**, 582-591 (2017).
22. Hall E. The deformation and ageing of mild steel: III discussion of results. *Proceedings of the Physical Society Section B* **64**, 747 (1951).
23. Li D, et al. Additive manufacturing of high strength near β titanium alloy Ti-55511 by engineering nanoscale secondary α laths via in-situ heat treatment. *Mater Sci Eng A* **814**, 141245 (2021).
24. Zhang T, et al. A new α + β Ti-alloy with refined microstructures and enhanced mechanical properties in the as-cast state. *Scr Mater* **207**, 114260 (2022).
25. Kocks U F, Mecking H. Physics and phenomenology of strain hardening: the FCC case. *Prog Mater Sci* **48**, 171-273 (2003).
26. de Formanoir C, et al. Micromechanical behavior and thermal stability of a dual-phase α+α' titanium alloy produced by additive manufacturing. *Acta Mater* **162**, 149-162 (2019).
27. Jang T J, et al. Shear band-driven precipitate dispersion for ultrastrong ductile medium-entropy alloys. *Nat Commun* **12**, 4703 (2021).
28. Mao Q, Zhang Y, Liu J, Zhao Y. Breaking Material Property Trade-offs via Macrodesign of Microstructure. *Nano Lett* **21**, 3191-3197 (2021).
29. Su J, et al. Revealing the decomposition mechanisms of dislocations and metastable α' phase and their effects on mechanical properties in a Ti-6Al-4V alloy. *J Mater Sci Technol* **107**, 136-148 (2021).
30. Pande C S, Cooper K P. Nanomechanics of Hall–Petch relationship in nanocrystalline materials. *Prog Mater Sci* **54**, 689-706 (2009).
31. Fan L, et al. Ultrahigh strength and ductility in newly developed materials with coherent nanolamellar architectures. *Nat Commun* **11**, 6240 (2020).
32. Caillard D, Couret A. The Hall–Petch law investigated by means of in situ straining experiments in lamellar TiAl and deformed Al. *Microsc Res Tech* **72**, 261-269 (2009).
33. Barriobero-Vila P, et al. Interface-mediated Twinning-induced Plasticity in A Fine Hexagonal Microstructure Generated By Additive Manufacturing. *Adv Mater*, e2105096 (2021).
34. Bahador A, et al. Ultrafine-grain formation and improved mechanical properties of novel extruded Ti-Fe-W alloys with complete solid solution of tungsten. *J Alloys Compd* **875**, 160031 (2021).
